# RPS3‐Enriched Extracellular Vesicles Mediate Liver‐Spinal Cord Inter‐Organ Communication

**DOI:** 10.1002/advs.202517019

**Published:** 2026-01-09

**Authors:** Peiwen Song, Zuomeng Wu, Yixiang Dong, Yunxiong Fang, Shiyu Bian, Daokuan Wang, Huang Fang, Yunlei Liu, Wang Ying, Jun Qian, Tianyu Han, Cailiang Shen

**Affiliations:** ^1^ Department of Orthopedics (Spinal Surgery) The First Affiliated Hospital of Anhui Medical University Laboratory of Spinal and Spinal Cord Injury Regeneration and Repair The First Affiliated Hospital of Anhui Medical University Hefei China; ^2^ Department of Orthopedics (Spinal Surgery) The First Affiliated Hospital of USTC Hefei China; ^3^ Department of Clinical Laboratory The First Affiliated Hospital of Anhui Medical University Hefei China; ^4^ Department of Radiology The First Affiliated Hospital of Anhui Medical University Hefei China

**Keywords:** astrocytes, axonal regeneration, extracellular vesicles, neural stem cells, spinal cord injuries

## Abstract

Spinal cord injury (SCI) induces bidirectional inter‐organ communication via extracellular vesicles (EVs) with multiple peripheral organs. Here, we identify the liver as a critical regulator that inhibits endogenous neuronal repair. Proteomics of plasma EVs from SCI patients and RNA‐sequence of post‐injury livers revealed a rapid increase of ribosomal protein S3 (RPS3) in plasma EVs and liver‐derived EVs (LEVs). These RPS3‐enriched LEVs are transported to the spinal cord lesion sites, where they are taken up by neural stem cells (NSCs) and astrocytes. Mechanistically, RPS3 activates nuclear factor kappa‐light‐chain‐enhancer of activated B cells (NF‐κB) signaling in recipient cells, inhibiting NSC differentiation into neurons and oligodendrocytes and polarizing astrocytes toward a neuroinflammatory phenotype. Further detection identified activated Kupffer cells (KCs) as the primary source of RPS3, initiating an intra‐hepatic cascade that further amplified RPS3 expression in hepatocytes. Crucially, in vivo depletion of KCs or hepatic RPS3 effectively attenuated NF‐κB activation, restored axonal regeneration and remyelination, and promoted neurological functional recovery. This work highlights a liver‐spinal cord axis wherein RPS3‐enriched hepatic KC‐derived EVs impair central nervous system (CNS) regeneration via the NF‐κB activation, presenting a promising prognostic biomarker and novel therapeutic target for SCI.

## Graphical abstract

Spinal cord injury activates the liver to send extracellular vesicles loaded with RPS3 protein to the lesion site. These vesicles are taken up by neural stem cells and astrocytes, triggering NF‐κB signaling, impairing the regeneration of neurons and myelin, and promotes harmful inflammation, ultimately hindering recovery. Blocking this liver‐spinal cord communication pathway presents a promising therapeutic strategy.

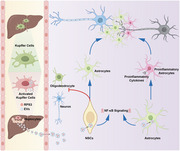

## 1 Introduction

Spinal cord injury (SCI) is a devastating neurological condition that leads to profound motor, sensory, and autonomic dysfunction [1, 2, 3]. Central nervous system (CNS) trauma, including SCI, shares many features that are often categorized into primary and secondary phases. The primary injury is defined by the acute damage to neural tissue resulting from mechanical forces and leads to largely irreversible cell loss. The secondary injury phase is characterized by delayed post‐trauma destructive inflammation, leading to further tissue damage over time and creating an unfavorable environment for neuronal regeneration [1, 4].

Extracellular vesicles (EVs) are membrane‐bound nanoparticles that act as carriers of diverse bioactive cargoes, including proteins, non‐coding RNAs, nucleic acids, and lipids, that can vary in response to stimuli [5, 6, 7, 8, 9, 10]. EVs have emerged as mediators of critical physiological functions in the CNS [7, 8, 10] and play a key role in the progression of the secondary injury phase of neurotrauma by transferring bioactive cargoes to recipient cells within the local CNS [10, 11, 12, 13, 14]. In addition to local communication, emerging evidence indicates that CNS trauma and neuroinflammation induce the release of EVs into circulation, which robustly activate the liver to mount the hepatic acute phase response, leading to the production of pro‐inflammatory cytokines and chemokines. These, in turn, activate the systemic immune system and accelerate the progression of CNS injury [10, 15, 16]. These studies highlight that CNS‐released EVs can modulate the functions of the recipient liver. However, the reverse pathway, specifically, whether and how the EVs derived from the SCI‐activated liver influence neuronal regeneration following SCI through inter‐organ communication, remains largely unclear.

**SCHEME 1:**
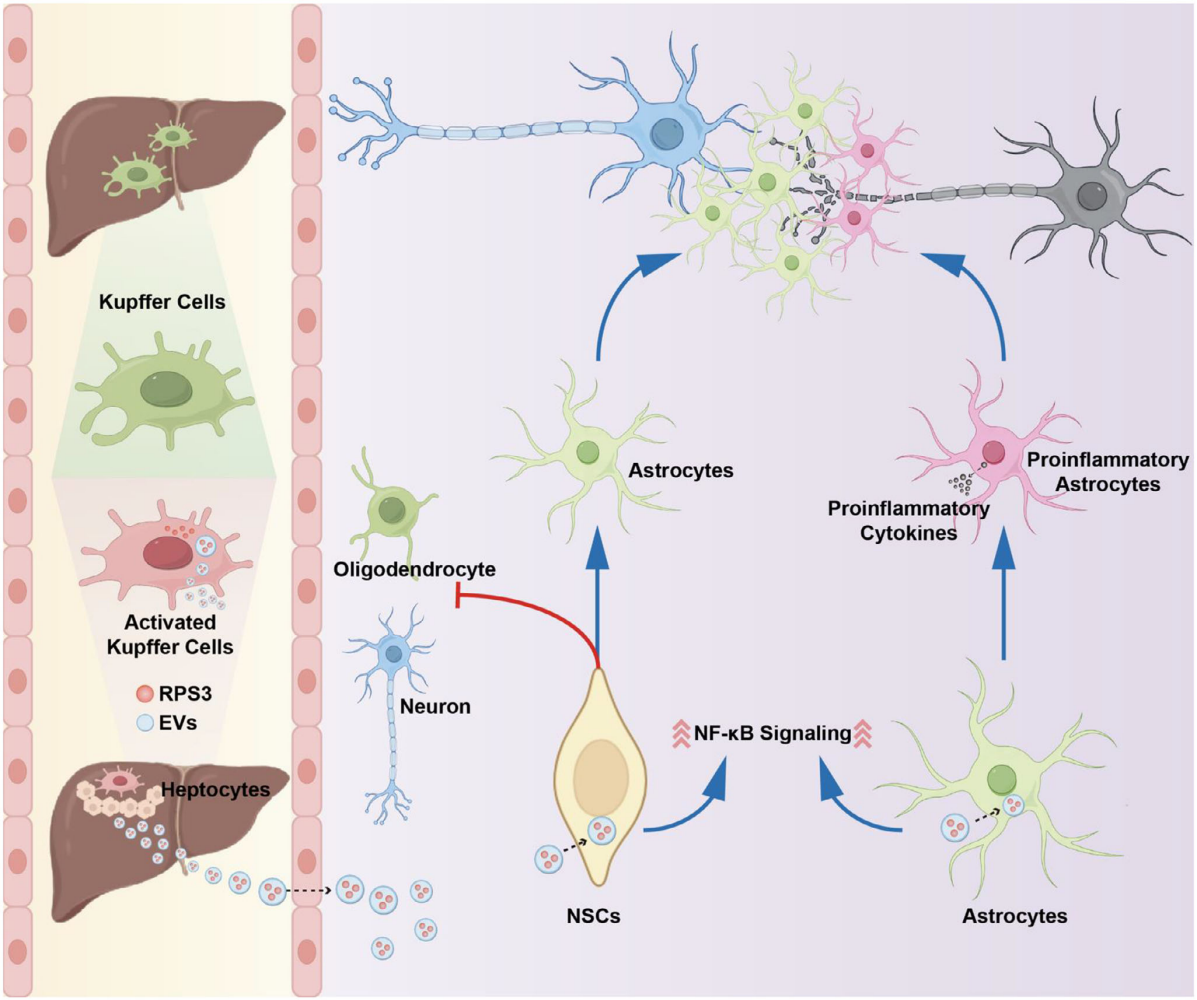
Schematic diagram of liver‐to‐spinal cord inter‐organ communication.

Here, we uncover a novel liver‐to‐spinal cord inter‐organ communication mediated by EVs that actively suppresses neuronal repair after SCI (Scheme 1). We demonstrate that EVs derived from post‐SCI liver are rapidly transported to the injured spinal cord via circulation and preferentially taken up by endogenous neural stem cells (NSCs) and astrocytes within the lesion sites. These EVs inhibit NSCs differentiation into neurons and oligodendrocytes and promote astrocytes polarization into neuroinflammatory reactive astrocytes. Further mass spectrometry (MS)‐based proteomics of plasma‐derived EVs from SCI patients and RNA‐sequencing of the post‐SCI liver revealed that SCI rapidly induces the enrichment of ribosomal protein S3 (RPS3) in liver‐derived EVs (LEVs), with activated Kupffer cells (KCs) identified as the primary source of RPS3. RPS3 induces the activation of nuclear factor kappa‐light‐chain‐enhancer of activated B cells (NF‐κB) signaling, which mediates the differentiation of NSCs and polarization of astrocytes at lesion sites. Depletion of RPS3 in KCs or the liver attenuates NF‐κB activation, promoting NSC differentiation into neurons and oligodendrocytes, preventing the activation of neuroinflammatory astrocytes, enhancing axonal regeneration and remyelination, and ultimately leading to significant functional recovery. Together, these findings indicate that LEVs play a key role in regulating the CNS regenerative microenvironment by carrying RPS3 after SCI and provide valuable insights into how EV‐based inter‐organ delivery affects the physiological processes in SCI.

## 2 Results

### 2.1 Liver EVs Alter Local Endogenous NSCs Differentiation

**FIGURE 1:**
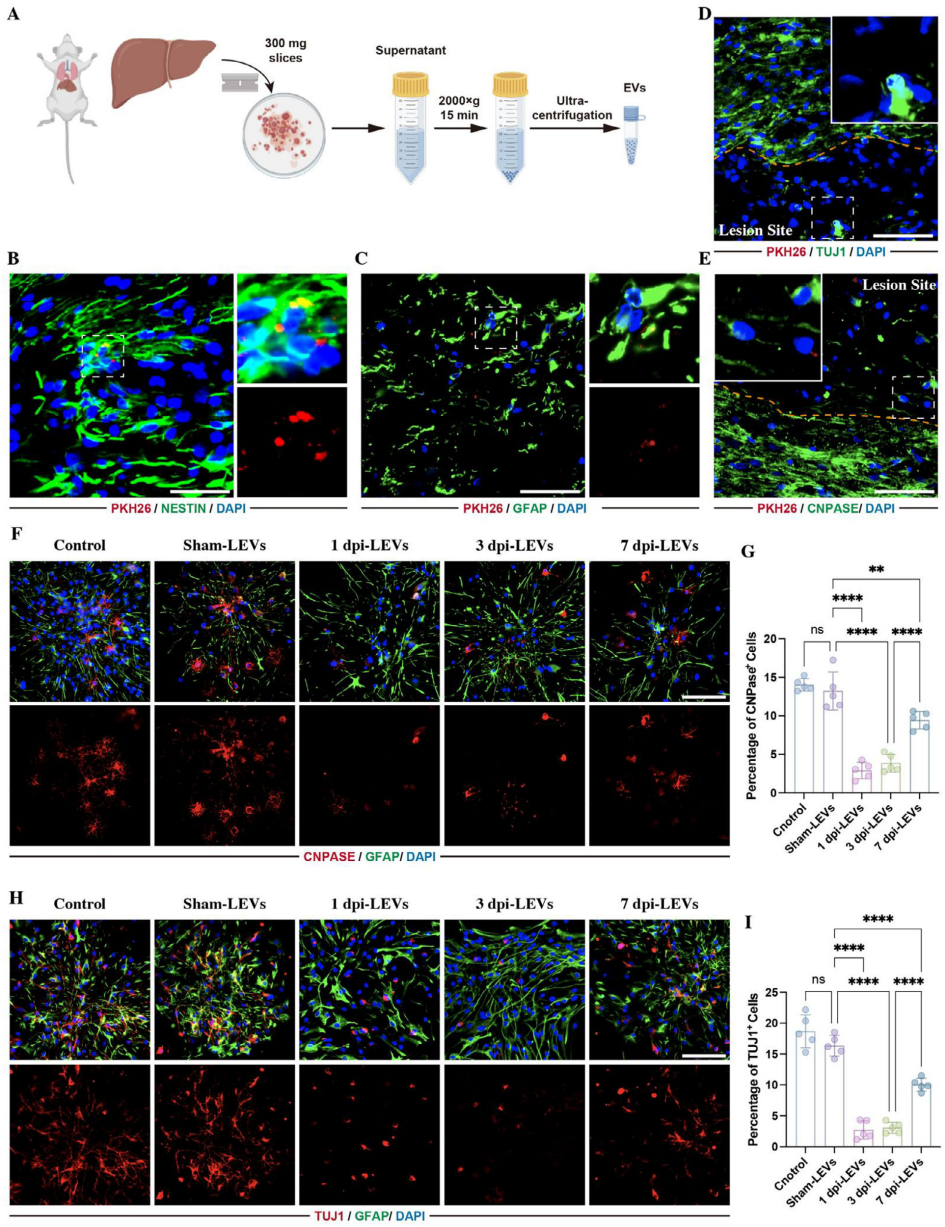
Postinjury LEVs regulated NSCs differentiation. (A) Schematic diagram of LEVs collection. (B–E) Immunostaining showing PKH‐26‐labeled LEVs (red) were taken up by Nestin‐positive NSCs (green) (B) or by GFAP‐positive astrocytes (green)(C) at lesion sites following 24 h post SCI. Seldom PKH‐26‐labeled LEVs were colocalized with TUJ1‐positive neurons (D) or CNPASE‐positive oligodendrocytes (E) (scale bars, 50 µm). (F,G) Representative images and related quantification of the percentage of CNPase‐positive cells in NSCs that were treated with 25 µL of sham‐LEVs, 1 dpi‐LEVs, 3 dpi‐LEVs, or 7 dpi‐LEVs for 5 days (*n* = 5, scale bars, 100 µm). (H,I) Representative images and related quantification of the percentage of TUJ1‐positive cells in NSCs treated with 25 µL different LEVs for 5 days (*n* = 5, scale bars, 100 µm). All data are presented as the mean ± standard deviation (SD). ns, *p*>0.05, ^*^ *p* < 0.05, ^**^ *p*<0.01, ^***^ *p*<0.001, ^****^ *p* <0.0001.

To investigate how the liver affects lesion sites following SCI, we collected LEVs at 1 day post‐injury (1 dpi) (Figure 1A). These collected LEVs were identified by transmission electron microscopy (Figure S1A), dynamic light scattering (Figure S1B), and western blotting (Figure S1C), then labeled with Polymeric Khlorin Hydrazine 26 (PKH 26). Subsequently, the PKH26‐labeled LEVs were injected into SCI rats through the tail vein, followed by immunostaining of spinal cord NSCs, astrocytes, neurons, and oligodendrocytes with markers NESTIN, glial fibrillary acidic protein (GFAP), β‐III‐tubulin (TUJ1), and 2',3'‐cyclic nucleotide 3' phosphodiesterase (CNPase) respectively. This showed that the PKH‐26 labeled LEVs were mainly taken up by the NESTIN‐positive NSCs (Figure 1B) and GFAP‐positive astrocytes (Figure 1C). In contrast, PKH‐26‐labeld EVs were rarely found in TUJ1‐positive neurons (Figure 1D) and CNPase‐positive oligodendrocytes (Figure 1E), owing to their sparse presence at the lesion sites.

As endogenous NSCs play a key role in the self‐ neurogenesis following SCI [17, 18, 19], the bioeffects of LEVs on the differentiation of NSCs were assessed. We collected LEVs at different time points following SCI and cultured them with NSCs. Immunostaining revealed that the percentage of CNPase‐positive oligodendrocytes and TUJ1‐positive neurons was significantly reduced by LEVs collected from SCI rats, specifically LEVs derived from rats at 1 and 3 day post‐injury (dpi) (1 dpi‐LEVs and 3 dpi‐LEVs) (Figure 1F–I), indicating that the LEVs prevented NSCs differentiation into oligodendrocytes and neurons.

### 2.2 RPS3 was Upregulated in LEVs, Blood, and Lesion Sites Postinjury

**FIGURE 2:**
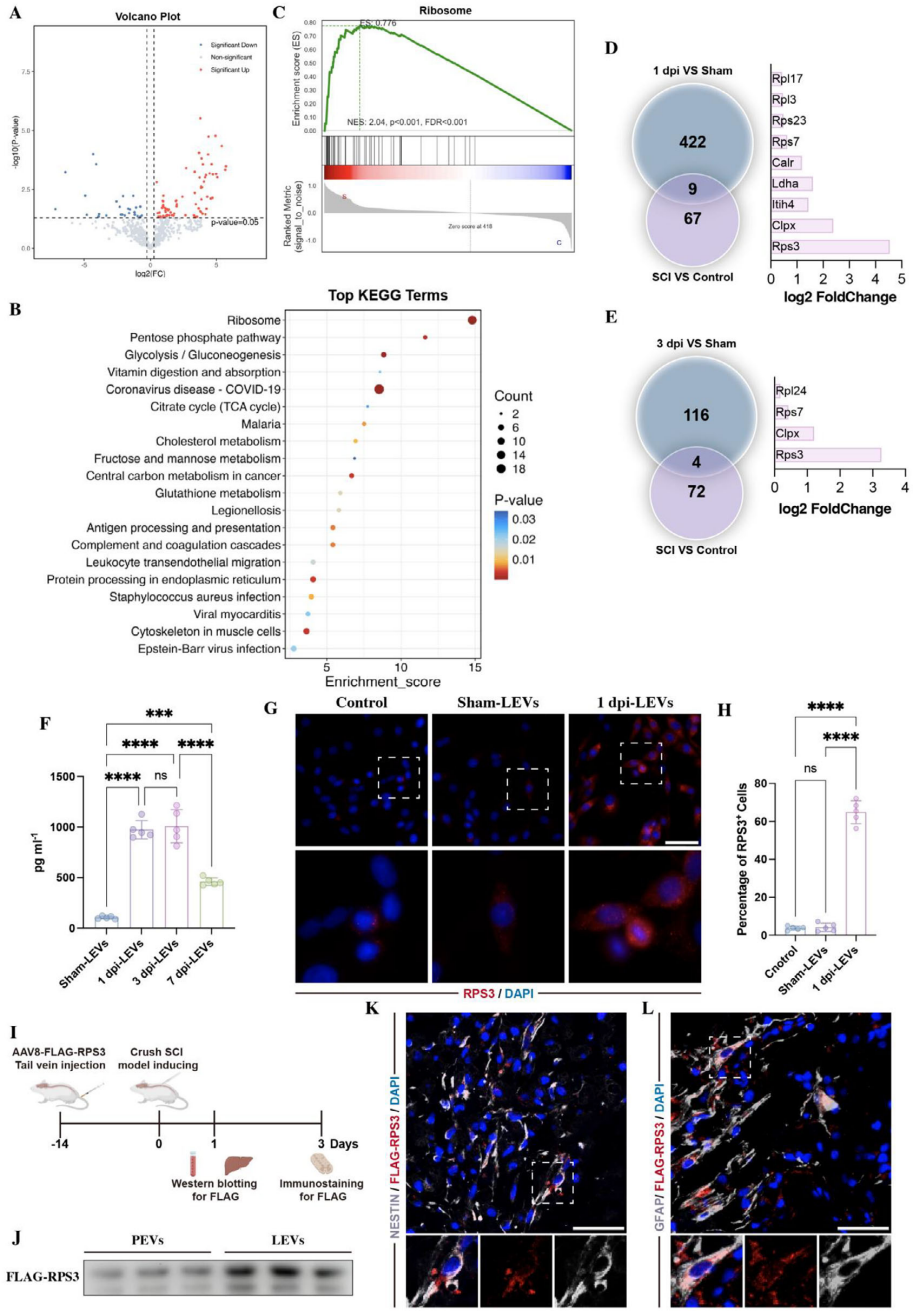
SCI enriches RPS3 in PEVs and LEVs. (A) Volcano plot showing differentially expressed proteins within PEVs from SCI (AIS A) patients (*n* = 3), compared to healthy controls (*n* = 3). (B) KEGG analysis revealing ribosome as the most significant enrichment in PEVs from SCI patients, compared to controls. (C) Gene set enrichment analysis of differentially expressed proteins within PEVs. (D,E) RNA‐sequence showing differentially expressed genes in 1 dpi livers (*n* = 3) (D) and 3 dpi livers (*n* = 3) (E), compared to sham groups. Venn diagram showing overlap between differentially expressed proteins in PEVs and differentially expressed genes in livers at 1 or 3 dpi. (F) Detection of RPS3 within LEVs at different time points postinjury by ELISA (*n* = 5). (G,H) Immunostaining of RPS3 in NSCs following the addition of 25 µL sham‐LEVs (*n* = 5) or 25 µL 1 dpi LEVs (*n* = 5) (scale bars, 50 µm). (I) Schematic of AAV8‐shRPS3 injection. (J) Western blotting confirming the presence of FLAG‐RPS3 in PEVs and LEVs (*n* = 3). (K,L) Immunostaining showing FLAG‐tagged RPS3 was localized within Nestin‐positive NSCs (K) and GFAP‐positive astrocytes (L) at lesion sites following 24 h post SCI. (scale bars, 50 µm). All data are presented as the mean ± standard deviation (SD). ns, *p*>0.05, ^*^ *p* <0.05, ^**^ *p*<0.01, ^***^ *p*<0.001, ^****^ *p* <0.0001.

To identify the LEV proteins responsible for mediating NSCs differentiation, we collected the EVs from plasma samples (plasma EVs, PEVs) of SCI patients (Figure S2A–D). Proteomics assessment revealed that 76 proteins were upregulated and 26 were downregulated in PEVs derived from SCI patients (Figure 2A). Kyoto encyclopedia of genes and genomes (KEGG) revealed that the most significant enrichment was found in the ribosome pathway (Figure 2B). Gene set enrichment analysis mapped 43 differentially expressed proteins to ribosome (Figure 2C; Figure S2E). Network analysis of protein‐protein interactions identified RPS3 as the top‐ranked protein (Figure S2F,G).

To further evaluate whether the alteration of cargoes in PEVs was related to the liver, we performed RNA sequencing on the liver at days 1 and 3 post‐SCI. At 1 dpi, 431 genes were upregulated in the liver, and combining these results with differentially expressed proteins in PEVs from SCI patients, nine proteins from these 431 altered genes were found to be upregulated in PEVs (Figure 2D). The most significantly altered gene in the liver was RPS3 (Figure 2D). At 3 dpi, 120 upregulated genes were identified in the liver, with 4 proteins from these altered genes enriched within PEVs, especially RPS3 (Figure 2E). Based on these data, we proposed that RPS3, a component of the small 40S ribosomal subunit that primarily associates with an initiation factor to facilitate ribosomal maturation and translation initiation [20, 21], might be responsible for the LEVs‐related bioeffects following SCI.

To confirm whether SCI induced the upregulation of PRS3 expression within LEVs, we examined RPS3 expression in LEVs following SCI. ELISA assay showed that RPS3 enrichment in LEVs was significantly increased following SCI, reaching a peak at 1 and 3 dpi (Figure 2F). Furthermore, immunostaining of NSCs treated with 1 dpi LEVs for 24 h revealed a marked rise in the proportion of RPS3‐positive cells (Figure 2G,H). These findings suggest that SCI promotes the enrichment of RPS3 in LEVs. To further investigate whether these LEVs carrying RPS3 are directly transported to the lesion site, we employed tail vein injection of adeno‐associated virus serotype 8 (AAV8), which has a strong affinity for liver cells, to target overexpress FLAG‐RPS3 in the liver (Figure 2I). Western blotting confirmed the presence of FLAG‐RPS3 in PEVs and LEVs (Figure 2J). Immunostaining for FLAG at lesion sites revealed that the presence of liver‐derived FLAG^+^ RPS3 in Nestin^+^ NSCs (Figure 2K) and GFAP^+^ astrocytes (Figure 2L), confirming direct transfer of RPS3 from the liver to the spinal cord lesion following SCI.

### 2.3 LEVs Carrying RPS3 Regulated NSC Differentiation Through Activating NF‐κB Signaling

**FIGURE 3:**
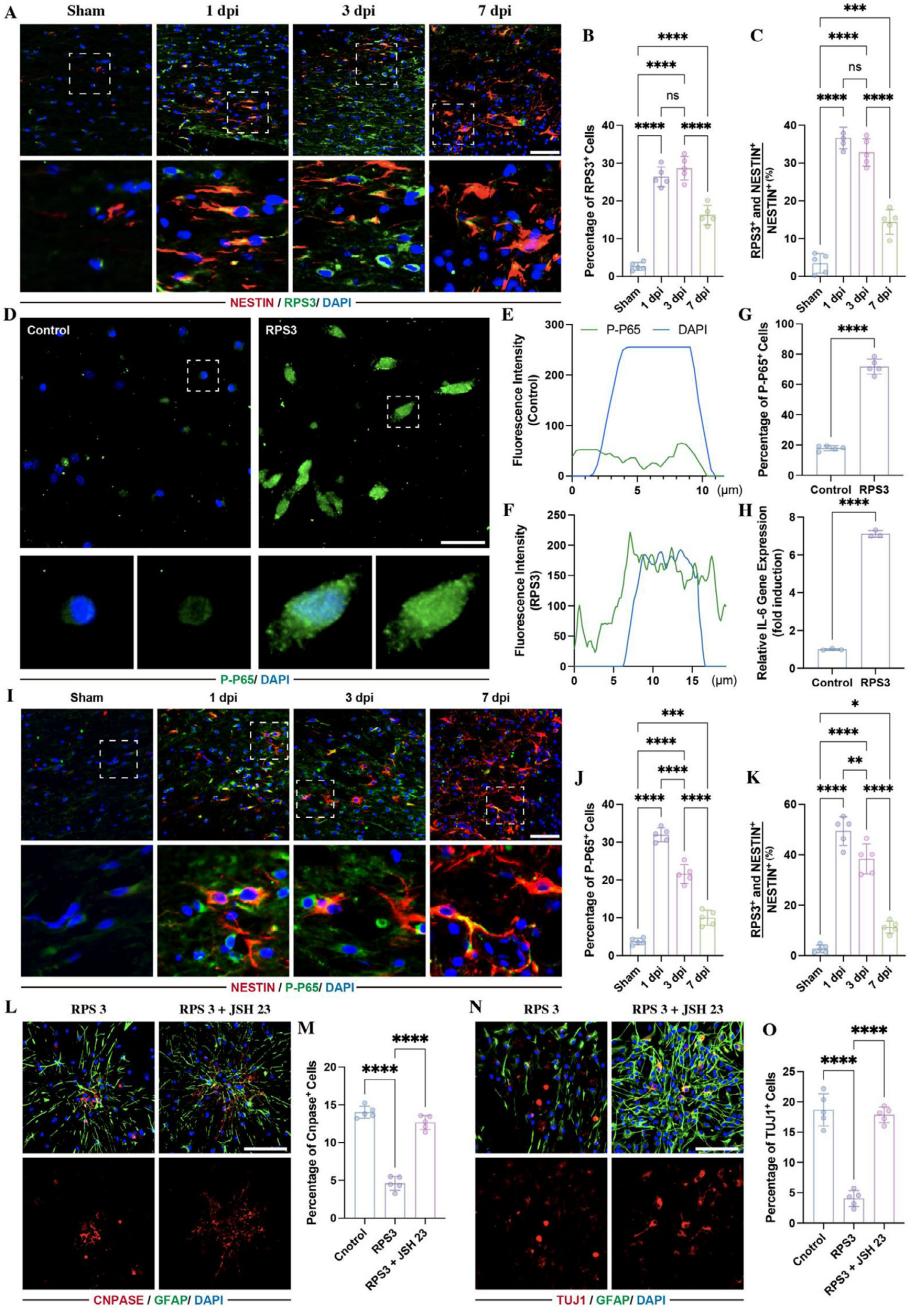
RPS3 regulated NSC differentiation via activating NF‐κB signaling. (A–C) Immunostaining of RPS3 and NESTIN within lesion sites at different time points following SCI (*n* = 5, scale bars, 100 µm). (D) Immunostaining of P‐P65 in NSC following 20 ng/mL RPS3 treatment (scale bars, 50 µm). (E,F) Fluorescence intensity of P‐P65 and DAPI in NSCs treated with or without RPS3. (G) Quantitative analysis of P‐P65‐positive cells (*n* = 5). (H). RT‐qPCR analysis of IL‐6 gene expression in NSCs treated with or without RPS3 (*n* = 3). (I–K) Immunostaining of P‐P65 and NESTIN within lesion sites at different time points following SCI (*n* = 5; scale bars, 100 µm). (L–O). Immunostaining detecting the percentage of CNPASE‐positive oligodendrocytes (L,M) and TUJ1‐positive neurons (N,O) in NSCs treated with RPS3 in the presence or absence of 30 µm JSH23 (*n* = 5; scale bars, 100 µm). All data are presented as the mean ± standard deviation (SD). ns, *p*>0.05, ^*^ *p* <0.05, ^**^ *p*<0.01, ^***^ *p*<0.001, ^****^ *p* <0.0001.

To investigate whether RPS3 affect NSC differentiation, we first detected the expression of RPS3 at lesion sites by immunostaining, finding that the percentage of RPS3‐positive cells increased following SCI (Figure 3A,B), and more than 30% of NESTIN‐positive NSCs were marked with RPS3 at 1 and 3 dpi (Figure 3A,C), indicating upregulation of RPS3 within the endogenous NSCs at lesion sites. Subsequently, we treated NSCs with RPS3. Since RPS3 is recognized as a functional subunit of NF‐κB that interacts with p65 subunit, which dramatically enhances the DNA‐binding affinity of NF‐κB complexes to specific κB sites, thus activating transcription of downstream genes [20, 22], the expression of the P‐P65 and its downstream gene (*Il‐6*) was detected. Immunostaining revealed that RPS3 treatment promoted P‐P65 translocation into the nucleus (Figure 3D–F) and increased the percentage of P‐P65‐positive cells (Figure 3D,G), accompanied by upregulation of *Il‐6* gene expression (Figure 3H). Consistent with RPS3 expression at lesion sites, P‐P65 expression levels were significantly increased in NSCs following SCI, reaching a peak at 1 dpi (Figure 3I–K). To detect the bioeffects of RPS3 on NSC differentiation, NSCs were cultured with RPS3, resulting in a reduction in the percentage of both CNPase‐ and TUJ1‐positive cells (Figure 3L–O). Moreover, these RPS3‐induced effects were abolished by (E)‐2‐fluoro‐4′‐methoxystilbene (JSH23), a nuclear translocation inhibitor of P65 (Figure 3L–O). These data indicated that RPS3 regulated NSC differentiation through the activation of NF‐κB signaling.

**FIGURE 4:**
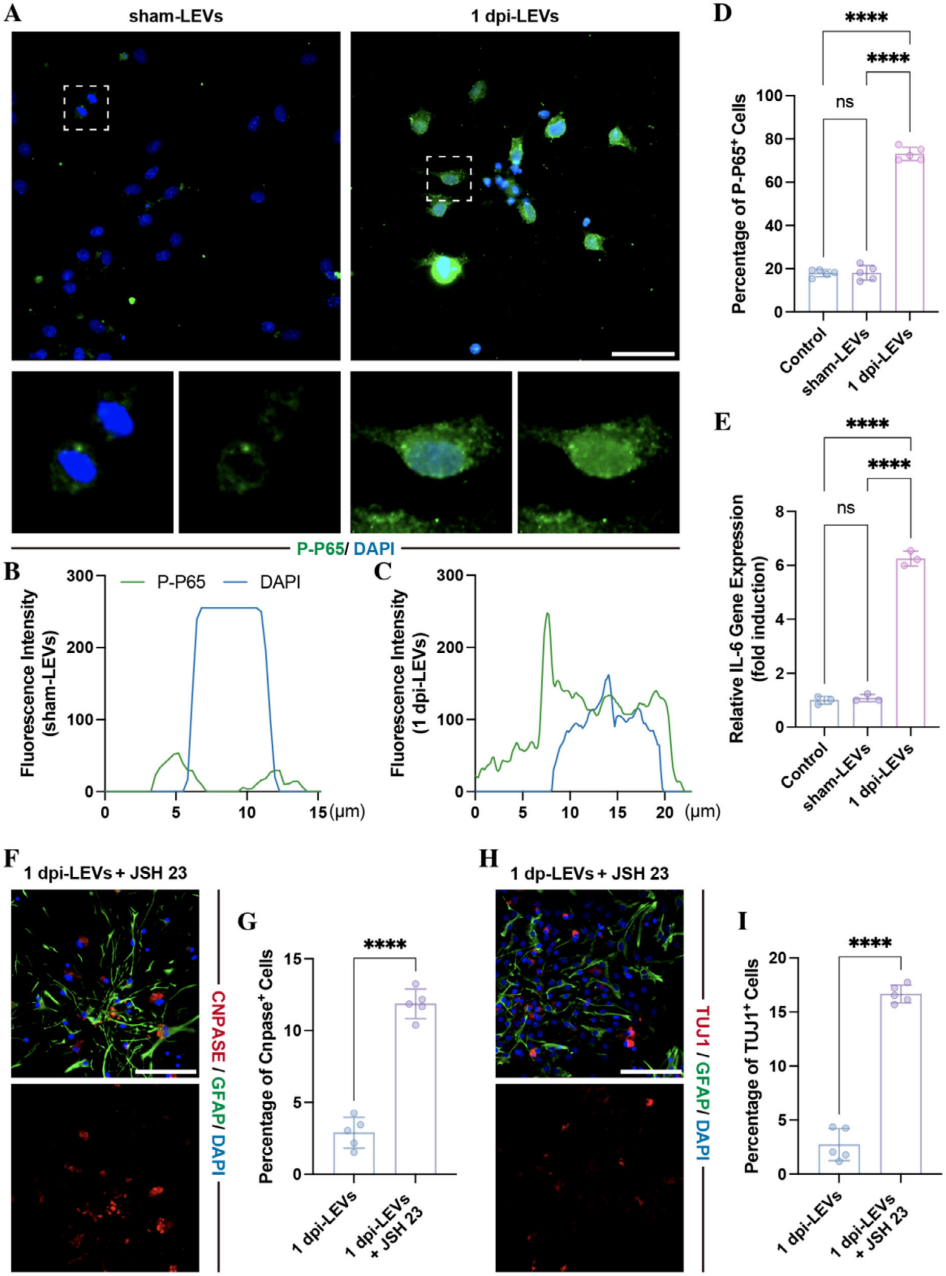
Postinjury LEVs regulated NSC differentiation via NF‐κB activation. (A) Immunostaining of P‐P65 in NSC following treatment with 25 µL 1 dpi LEVs or sham LEVs treatment (scale bars, 100 µm). (B,C) Fluorescence intensity of P‐P65 and DAPI in NSCs treated with 1 dpi LEVs or sham LEVs. (D) Quantitative analysis of P‐P65‐positive cells (*n* = 5). E) RT‐qPCR analysis of IL‐6 gene expression in NSCs (*n* = 3). (F–I) Immunostaining detecting the percentage of CNPASE‐positive oligodendrocytes (F,G) and TUJ1‐positive neurons (H,I) in NSCs treated with 1 dpi LEVs in the presence of 30 µM JSH23 (*n* = 5; scale bars, 100 µm). All data are presented as the mean ± standard deviation (SD). ns, *p*>0.05, ^*^ *p* <0.05, ^**^ *p*<0.01, ^***^ *p*<0.001, ^****^ *p* <0.0001.

To further confirm whether the enrichment of RPS3 in LEVs was associated with the altered effects on NSC differentiation, we treated NSCs with LEVs derived from sham rats (sham‐LEVs) or 1 dpi rats (1 dpi‐LEVs) and examined P‐P65 expression by immunostaining. Compared with control groups, sham‐LEVs did not induce nuclear translocation of P‐P65 (Figure 4A,B) or increase the percentage of P‐P65 positive cells (Figure 4A,D). In contrast, 1 dpi‐LEVs promoted nuclear translocation of P‐P65 and increased the percentage of P‐P65 positive cells (Figure 4A,C,D). PCR analysis revealed that the IL‐6 gene expression was upregulated by 1 dpi‐LEVs, but not by sham‐LEVs (Figure 4E). Furthermore, addition of JSH23 to NSCs in the presence of 1 dpi‐LEVs suppressed the 1 dpi‐LEVs‐induced effects on NSC differentiation (Figure 4F–I). These findings indicate that liver‐derived RPS3 regulates NSC differentiation through activation of NF‐κB signaling.

### 2.4 KCs Drive Liver‐Induced Inter‐Organ RPS3 Delivery

**FIGURE 5:**
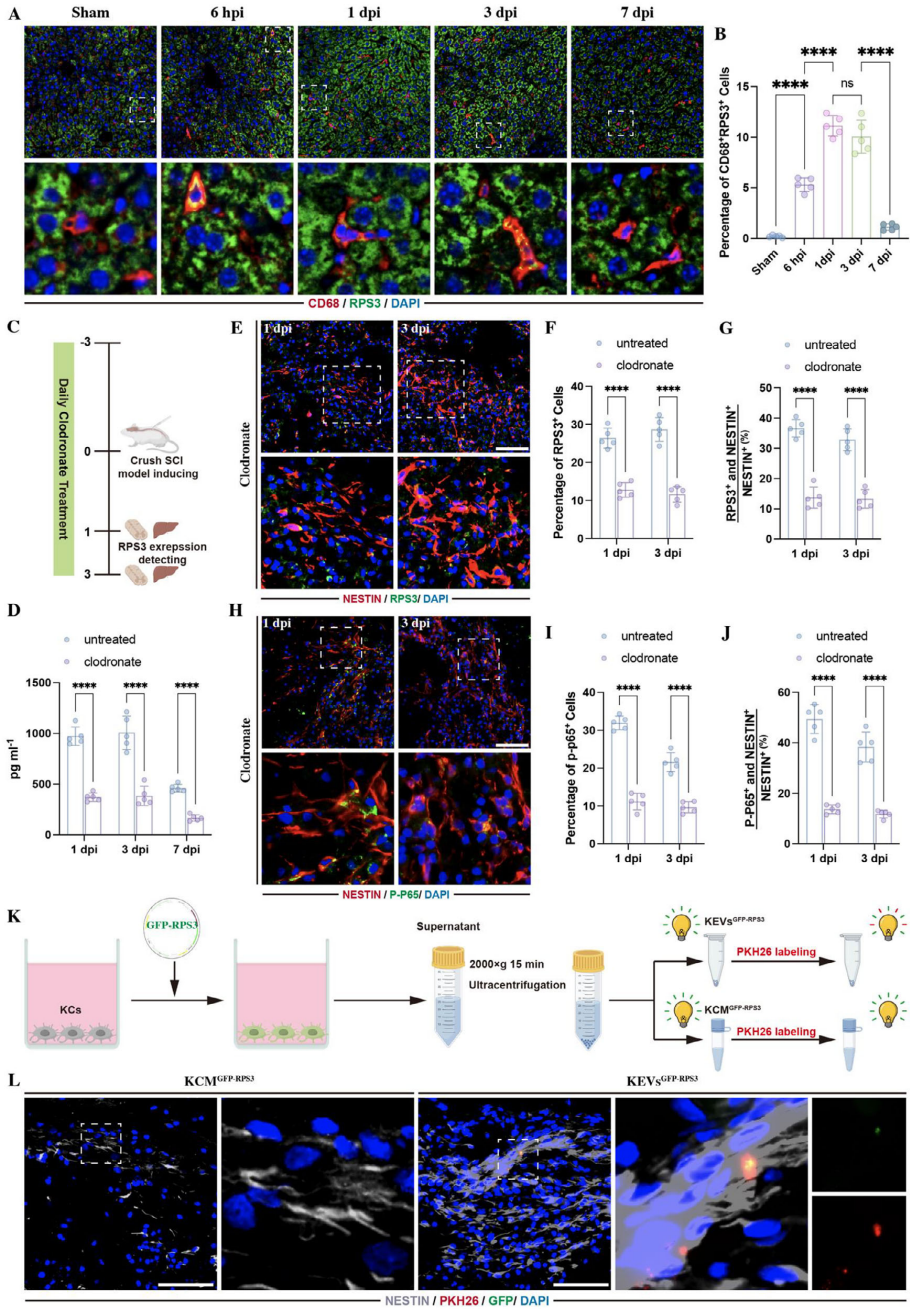
Kupffer cells drive inter‐organ RPS3 delivery. (A,B) Immunostaining detecting RPS3 in CD68‐positive macrophages in liver at different time points following SCI (*n* = 5, scale bars, 100 µm). (C) Schematic of clodronate treatment. (D) ELISA detecting RPS3 expression within LEVs at different time points postinjury (*n* = 5). (E–G) Immunostaining detecting RPS3 in NSCs within lesion sites at 1 and 3 dpi following clodronate injection (*n* = 5; scale bars, 100 µm). (H–J) Immunostaining detecting P‐P65 in NSCs within lesion sites at 1 and 3 dpi following clodronate injection (*n* = 5; scale bars, 100 µm). (K) Schematic of PKH‐26‐labeled KEVs^GFP‐RPS3^ and KCMs^GFP‐RPS3^ collection. (L) Immunostaining showing PKH‐26‐ and GFP‐RPS3 co‐labeled KEVs were taken up by Nestin‐positive NSCs (scale bars, 50 µm). All data are presented as the mean ± standard deviation (SD). ns, *p*>0.05, ^*^ *p* <0.05, ^**^ *p*<0.01, ^***^ *p*<0.001, ^****^ *p* <0.0001.

Hepatic macrophages are essential for mediating liver immunity and maintain hepatic homeostasis [23, 24, 25, 26]. We therefore hypothesized that the activated hepatic macrophages might contribute to the upregulation of RPS3 in liver. To confirm this hypothesis, we first detected the expression of RPS3 in hepatic macrophages. Immunostaining revealed that the expression of RPS3 was increased rapidly in the CD 68^+^ hepatic macrophages as early as 6‐h post‐injury (hpi) and reached a peak at 1 and 3 dpi (Figure 5A,B). Subsequently, we depleted hepatic macrophages in rats by intravenous injection of clodronate (Figure 5C). Compared with the untreated SCI rats, clodronate injection markedly reduced the RPS3 expression in LEVs (Figure 5D). Immunostaining showed that hepatic macrophages depletion in the liver reduced both RPS3(Figure 5E–G) and P‐P65 (Figure 5H–J) expression within lesion sites at 1 and 3 dpi. To determine whether hepatic macrophages ‐derived RPS3 could directly transfer to lesion sites following SCI, we cultured primary Kupffer cells, which are resident hepatic macrophages, and transfected them with exogenous RPS3, tagged with green fluorescent protein (GFP). Next, we collected EVs from GFP‐RPS3‐overexpressing KCs (KEVs^GFP‐RPS3^), followed by PKH‐26 labeling (Figure 5K). Fluorescence microscopy revealed co‐expression of GFP‐RPS3 and PKH‐26 in a cluster of EVs (Figure S3A). Additionally, to determine whether non‐EVs secreted factors could achieve inter‐organ delivery from the liver to lesion sites, we collected EV‐depleted conditioned medium from GFP‐RPS3‐overexpressing KCs (KCM^GFP‐RPS3^) (Figure 5K). In vitro, we treated NSCs with PKH26‐labeled KEVs^GFP‐RPS3^ or KCM^GFP‐RPS3^ for 6 h, finding that GFP was co‐localized with PKH26 within the cytoplasm of NSCs. In contrast, the non‐EV KCM^GFP‐RPS3^ treatment only induced the presence of GFP‐RPS3 within the cytoplasm (Figure S3B). Subsequently, we injected KEVs^GFP‐RPS3^ and KCM^GFP‐RPS3^ into SCI rats, respectively. After 24 h, GFP‐ and PKH‐26 double‐positive KEVs^GFP‐RPS3^ were found in NESTIN‐positive NSCs at lesion sites (Figure 5L). However, no GFP‐labeled RPS3 was noted at lesion sites in the rats that received KCM^GFP‐RPS3^ injection (Figure 5L). These data indicated that the EV membrane plays an important role in liver‐spinal cord long‐distance inter‐organ delivery of RPS3.

**FIGURE 6:**
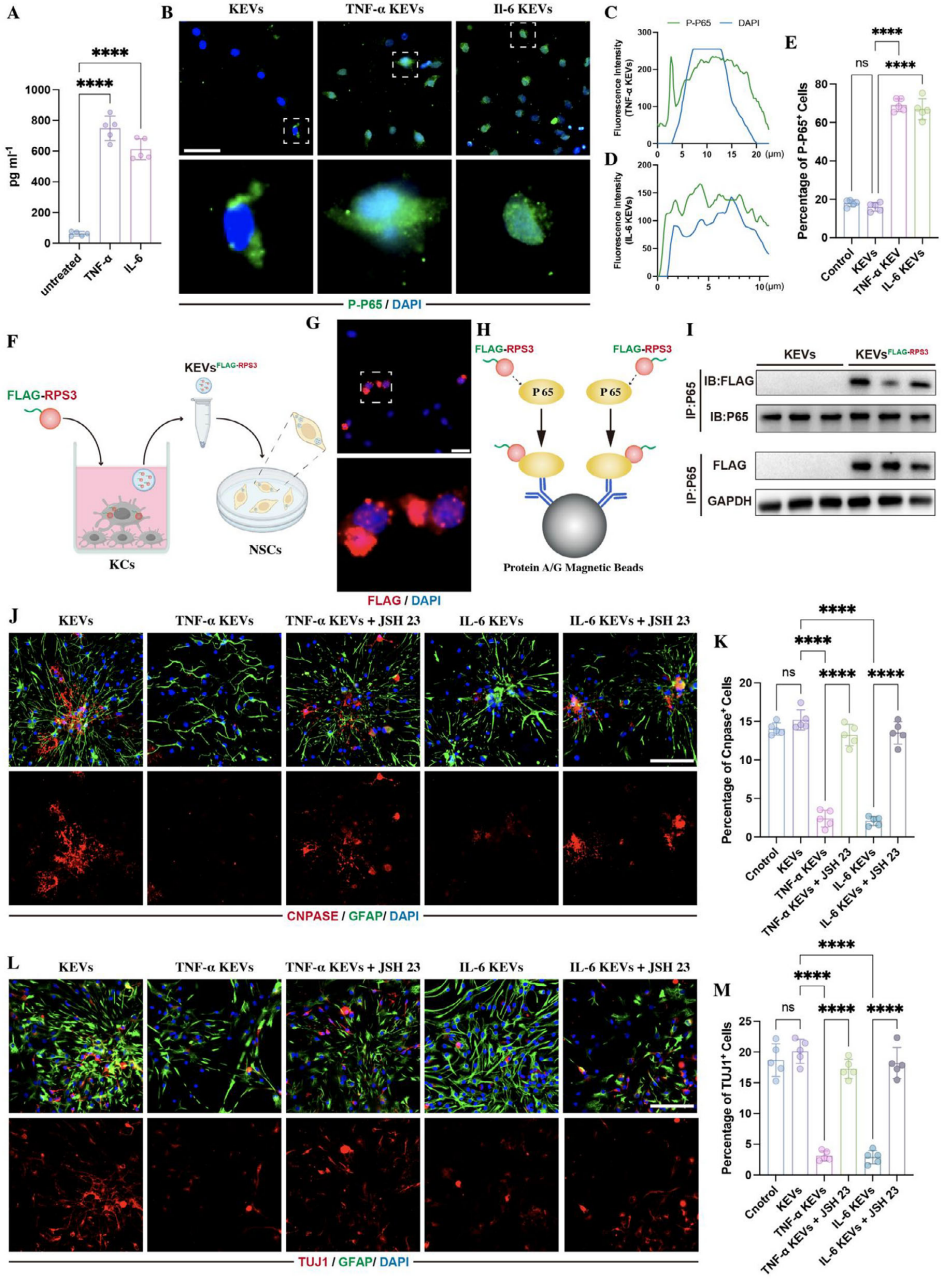
EVs derived from inflammation‐stimulated KCs mediated NSC differentiation through RPS3/NF‐κB activation. (A) ELISA detecting the expression of RPS in TNF‐α KEVs and IL‐6‐KEVs (*n* = 5); (B–E) Immunostaining of P‐P65 in NSC following the treatment with 25 µL TNF‐α KEVs or 25 µL IL‐6‐KEVs (*n* = 5; scale bars, 100 µm). (F) Schematic of FLAG‐labeled KEVs^FLAG‐RPS3^ collection. (G) Fluorescence microscopy showing the expression of FLAG‐PRS3 (red) within NSCs following the addition of KEVs^FLAG‐RPS3^ (scale bars, 25 µm). (H) Schematic of immunoprecipitation for the detection of FLAG‐RPS3. (I) immunoblot using FLAG antibody confirmed its interaction with P65 (*n* = 3). (J–M) Immunostaining detecting the percentage of CNPASE‐positive oligodendrocytes (J,K) and TUJ1‐positive neurons (L,M) in NSCs treated with TNF‐α KEVs‐ or IL‐6‐KEVs in the presence or absence of 30 µm JSH23 (*n* = 5; scale bars, 100 µm). All data are presented as the mean ± standard deviation (SD). ns, *p*>0.05, ^*^ *p* <0.05, ^**^ *p*<0.01, ^***^ *p*<0.001, ^****^ *p* <0.0001.

To investigate whether KEVs inhibited NSC differentiation into neurons and oligodendrocytes through NF‐κB signaling, we cultured NSCs with KEVs. Since CNS injury robustly activates the systemic immune system, leading to the upregulation of pro‐inflammatory cytokines in the liver [10, 27], we pretreated KCs with 25 ng/mL tumor necrosis factor alpha (TNF‐α) or 50 ng/mL IL‐6, followed by the collection of KEVs. Compared with control or non‐pretreated KEVs, the EVs derived from TNF‐α‐ or IL‐6‐stimulated KCs (TNF‐α KEVs or IL‐6 KEVs) significantly increased the expression of PRS3 in KCs (Figure S4A,B) and the enrichment of RPS3 in EVs (Figure 4A). The detection of P‐P65 expression by immunostaining revealed that the addition of TNF‐α‐ or IL‐6‐KEVs to NSCs markedly increased the percentage of P‐P65 positive cells and promoted P‐P65 translocation into the nucleus (Figure 6B–E). To further confirm whether these exogenous RPS3 could interacted with p65 in NSCs, we transfected KCs with exogenous RPS3, tagged with FLAG. Then, the EVs were collected from these FLAG‐RPS3‐transfected KCs (KEVs^FLAG‐RPS3^) and added to NSCs (Figure 6F). Immunostaining showed the FLAG‐positive RPS3 were localized within the cytoplasm, confirming these KCs‐derived FLAG‐RPS3 were taken up by NSCs (Figure 6G). Subsequently, a pull‐down assay using P65 antibody demonstrated its interaction with FLAG‐RPS3 in KEVs^FLAG‐RPS3^ treated NSCs (Figure 6H,I). In addition, evaluation of NSC differentiation revealed that the treatment of TNF‐α‐ or IL‐6‐ KEVs significantly reduced the percentage of neurons and oligodendrocytes (Figure 6J–M). The addition of JSH23 markedly repressed these TNF‐α‐ or IL‐6‐ KCs‐induced bioeffects on NSC differentiation, increasing the proportion of neurons and oligodendrocytes (Figure 6J–M). These data indicated that pro‐inflammatory cytokine stimulation enriched the RPS3 within KEVs, which regulated NSC differentiation by activating NF‐κB signaling.

**FIGURE 7:**
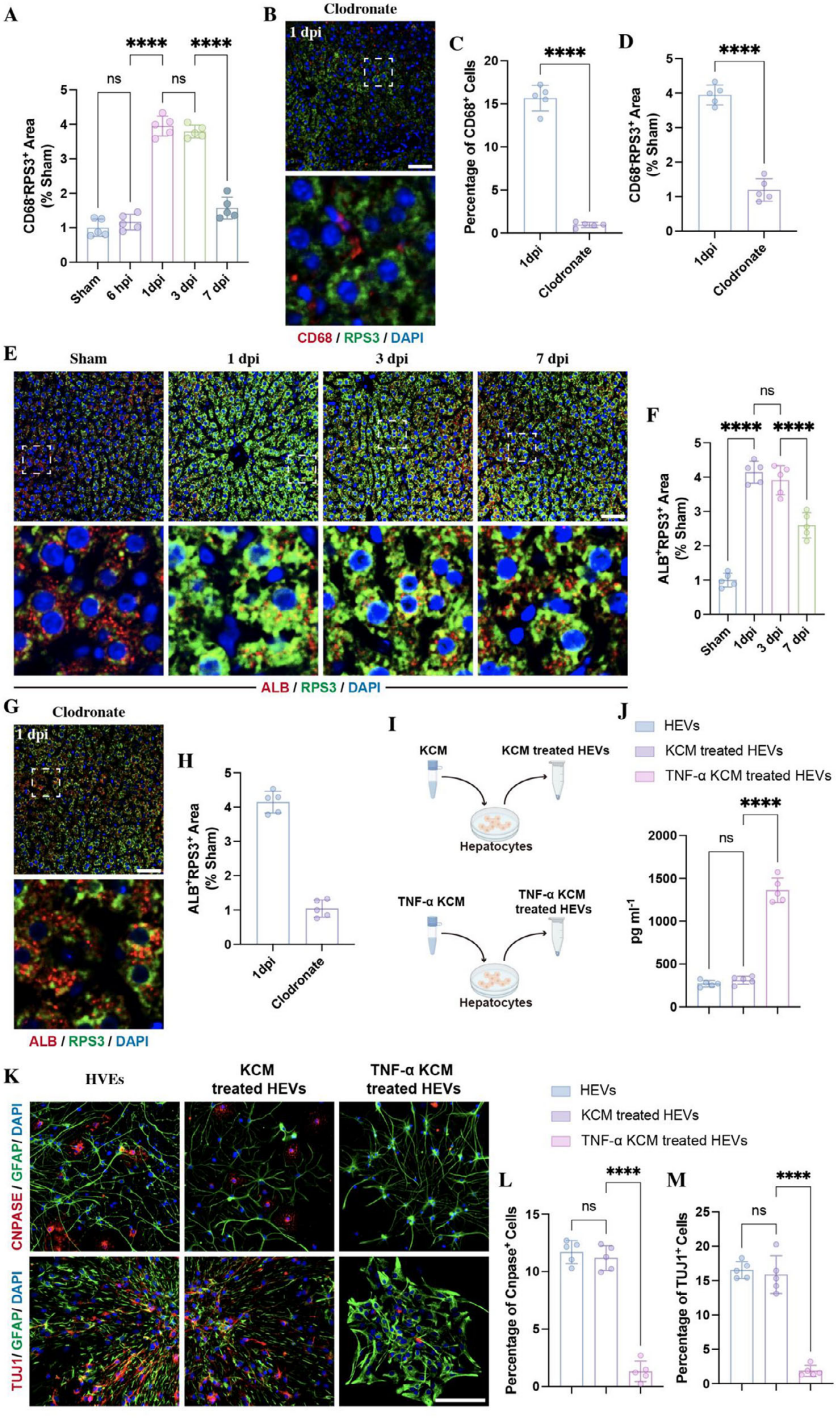
Activated KCs promote RPS3 enrichment in hepatocyte‐derived EVs. (A) Quantification of the percentage of CD68^−^RPS3^+^ areas (*n* = 5). (B–D) Immunostaining detecting RPS3 in CD68^+^ and CD68^−^ cells in liver at 1 dpi following clodronate injection (*n* = 5; scale bars, 100 µm). (E,F) Immunostaining detecting RPS3 in ALB^+^ hepatocytes in liver at different time points following SCI (*n* = 5; scale bars, 100 µm). (G,H) Immunostaining detecting RPS3 in ALB^+^ hepatocytes in liver at 1 dpi following clodronate injection (*n* = 5; scale bars, 100 µm). (I) Schematic of KCM‐treated HEVs collection. (J) ELISA detecting the expression of RPS in KCM‐treated HEVs (*n* = 5). (K–M) Immunostaining detecting the percentage of CNPASE‐positive oligodendrocytes and TUJ1‐positive neurons in NSCs treated with HEVs, KCM‐treated HEVs or TNF‐α KCM‐treated HEVs (*n* = 5; scale bars, 100 µm). All data are presented as the mean ± standard deviation (SD). ns, *p*>0.05, ^*^ *p* <0.05, ^**^ *p*<0.01, ^***^ *p*<0.001, ^****^ *p* <0.0001.

Interestingly, the immunostaining of RP3 in liver revealed that, in Sham groups, RPS3 expression was primarily localized to CD68^−^ cells (Figure 5A). In contrast to the rapid enrichment of RPS3 in CD68^+^ cells at 6 hpi, the expression level in CD68^−^ cells remained unaltered at this time point (Figures 5A,B and 7A). However, a delayed increase in RPS3 expression was detected in these CD68^−^ cells at 1 dpi. Furthermore, depletion of hepatic macrophages significantly abolished the upregulation of RPS3 in CD68^−^ cells at 1 dpi (Figure 7B–D). These findings suggest that hepatic macrophages may play a critical role in initiating RPS3 upregulation in the liver. Since hepatocytes are the main epithelial cells of the liver parenchyma, constituting around 80% of the liver volume and 60% of the liver cell population [28], we next detected the expression of RPS3 in hepatocytes. As expected, immunostaining for albumin (ALB) revealed that a significant RPS3 increase in ALB^+^ hepatocytes, which peaked at 1 and 3 dpi (Figure 7E,F). Moreover, these upregulation of RPS3 in hepatocytes was markedly abolished by the treatment of clodronate (Figure 7G,H). To further investigate whether activated KCs could promote RPS3 enrichment in hepatocytes, we treated hepatocytes with the conditioned medium from TNF‐α‐pretreated KCs (KCM) and then collected these hepatocytes‐derived EVs (HEVs) (Figure 7I). ELISA analysis of RPS3 expression in HEVs showed that treatment with TNF‑α‑KCM enhanced RPS3 levels in HEVs compared with treatment with KCM alone (Figure 7J). Evaluation of NSC differentiation revealed that the treatment of TNF‐α KCM treated HEVs significantly reduced the percentage of oligodendrocytes and neurons (Figure 7K,M). These data indicated that the activated KCs‐ mediated RPS3 enrichment in hepatocytes and its subsequent packaging into EVs suppress neuronal and oligodendrocytic differentiation of NSCs.

### 2.5 Knockdown of RPS3 in the Liver Attenuated the Activation of NF‐κB Signaling in NSCs

**FIGURE 8:**
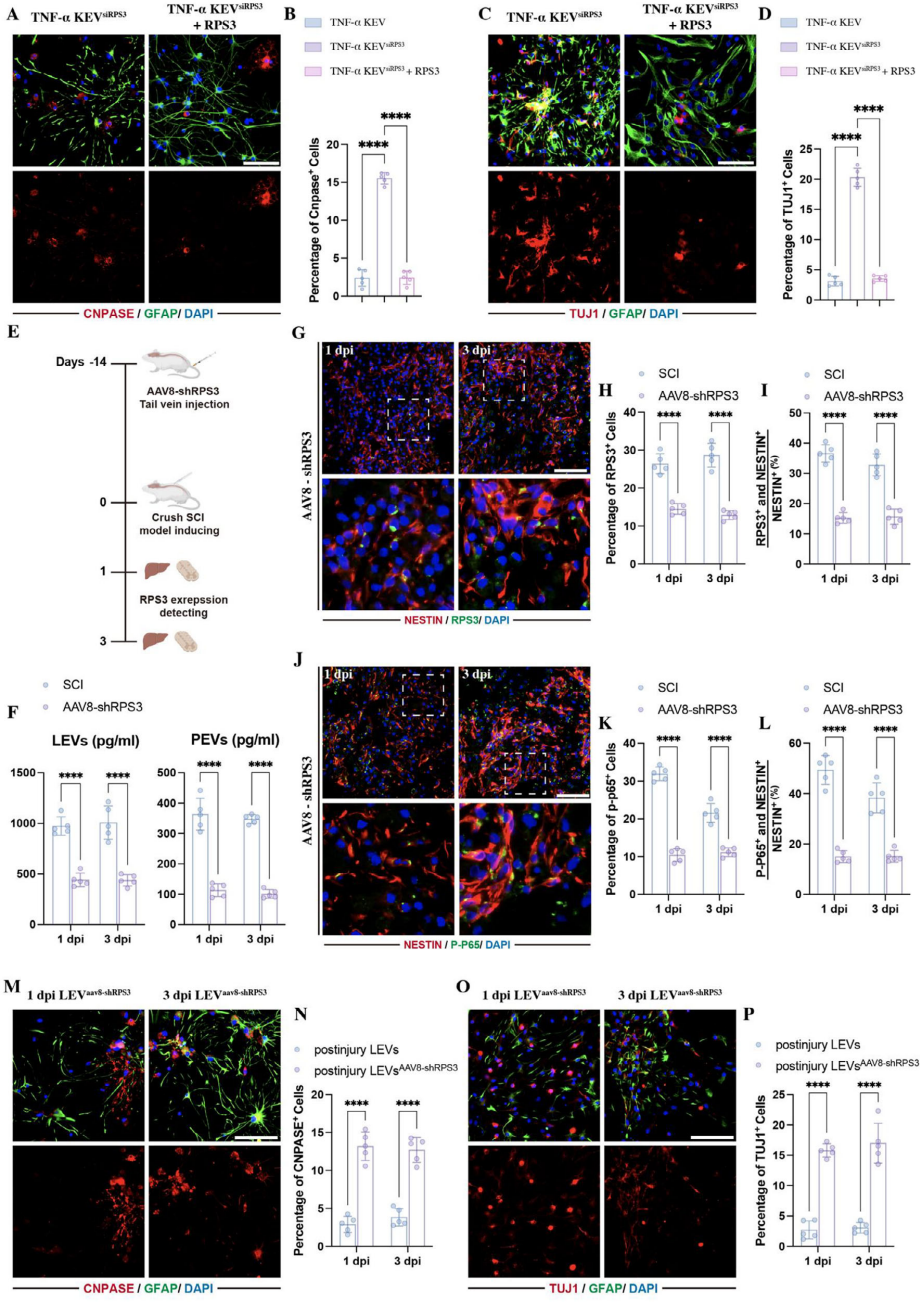
RPS3 played a critical role in Liver/KCs‐induced regulation of NSC differentiation. (A–D) Immunostaining detecting the percentage of CNPASE‐positive oligodendrocytes (A,B) and TUJ1‐positive neurons (C,D) in NSCs treated with 25 µL TNF‐α KEVs^siRPS3^, with or without exogenous RPS3 supplementation (*n* = 5; scale bars, 100 µm). (E) Schematic of AAV8‐shRPS3 injection. (F) ELISA detecting RPS3 expression in LEVs and PEVs following the AAV8‐shRPS3 injection (*n* = 5). (G–L) Immunostaining of RPS3 (G–I) and P‐P65 (J–L) in NSCs within lesion sites following the AAV8‐shRPS3 injection (*n *= 5; scale bars, 100 µm). (M–P) Immunostaining detecting the percentage of CNPASE‐positive oligodendrocytes (M,N) and TUJ1‐positive neurons (O,P) in NSCs treated with 25 µL LEVs^AAV8‐shRPS3^(*n* = 5; scale bars, 100 µm). All data are presented as the mean ± standard deviation (SD). ns, *p*>0.05, ^*^ *p* < 0.05, ^**^ *p*<0.01, ^***^ *p*<0.001, ^****^ *p* <0.0001.

To test whether KC‐derived RPS3 played a critical role in mediating NSC differentiation, we knocked down RPS3 in KCs by siRNA. The efficiency of RPS3 depletion in KCs was confirmed by immunofluorescence (Figure S5A). Then, EVs were collected from these RPS3‐deleted KCs in the presence of TNF‐α (TNF‐α‐KEV^siRPS3^). Detecting RPS3 expression by ELISA revealed that the enrichment of RPS3 was markedly reduced in TNF‐α‐KEV^siRPS3^ (Figure S5B). Subsequently, the bioeffects of these TNF‐α‐KEV^siRPS3^ on NSCs differentiation were detected. Immunostaining revealed that the depletion of RPS3 significantly abolished the TNF‐α‐KEVs‐induced effects, increasing the percentage of oligodendrocytes (Figure 8A,B) and neurons (Figure 8C,D). This effect was rescued by exogenous RPS3 supplementation (Figure 8A–D).

To target knockdown RPS3 expression in the liver, AAV 8 was applied to carry short hairpin RNA to knock down RPS3 in the liver by tail vein injection (Figure 8E). The detection of RPS3 expression within LEVs and PEVs by ELISA was performed to evaluate the efficiency of AAV8‐shRPS3 in the liver following SCI, showing that the injection of AAV8‐shRPS3 markedly decreases the enrichment of RPS3 within LEVs and PEVs at 1 and 3 dpi (Figure 8F). Additionally, immunostaining showed that the depletion of RPS3 in the liver also reduced the expression of RPS and P‐P65 in the NESTIN‐positive NSCs (Figure 8G–L). These data indicated that the LEVs influenced the activation of NF‐κB signaling at lesion sites through inter‐organ delivery of RPS3. In addition, we collected the LEVs at 1 and 3 dpi from the AAV8‐shRPS3 injected rats and treated NSCs with these LEVs, showing that the RPS3 knockdown in liver significantly abolished the 1 and 3 dpi LEVs‐related effects on mediating NSC differentiation, leading to an increased percentage of oligodendrocytes (Figure 8M,N) and neurons (Figure 8O,P).

### 2.6 Liver's Inter‐Organ Delivery of RPS3 Drives the Activation of Astrocytes

**FIGURE 9:**
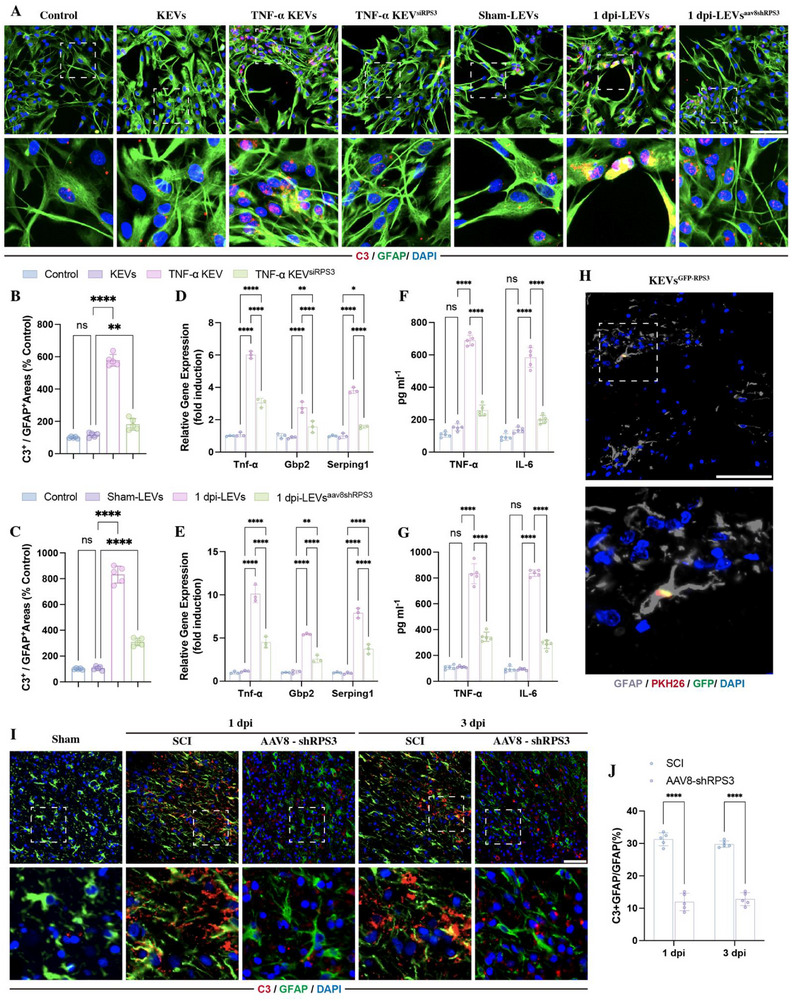
Livers/KC3 delivery of RPS3 regulated the polarization of astrocytes. (A–C) Immunostaining of C3 in GFAP‐positive astrocytes treated with different groups of KEVs or LEVs (*n* = 5; scale bars, 100 µm). (D,E) RT‐qPCR analysis of A1‐related gene expression in astrocytes treated with different KEVs or LEVs (*n* = 3). (F,G) ELISA detecting the expression of pro‐inflammatory cytokines in conditioned medium of KEVs‐ or LEVs‐treated astrocytes (*n* = 5). (H) Immunostaining showing co‐localization of GFP‐RPS3, PKH‐26‐labeled KEVs, and GFAP‐positive astrocytes in lesion sites 24 h following PKH‐26‐labeled KEVs and GFP‐RPS3 (scale bars, 100 µm). (I,J) Immunostaining and quantification of C3 in astrocytes at lesion sites at 1 and 3 dpi following AAV8‐shRPS3 injection (*n* = 5, scale bars, 100 µm). All data are presented as the mean ± standard deviation (SD). ns, *p*>0.05, ^*^ *p* <0.05, ^**^ *p*<0.01, ^***^ *p*<0.001, ^****^ *p* <0.0001.

Given that the LEVs (Figure 1E) and liver‐derived RPS3 (Figure 2L) were taken up by astrocytes, we first detected whether these EVs affected the activation of astrocytes in vitro by immunostaining with marker C3, which is the most commonly used specific marker for A1 neuroinflammatory reactive astrocytes [29, 30, 31]. It revealed that the treatment of TNF‐α KEVs and 1 dpi‐LEVs markedly increased the expression of C3 (Figure 9A–C) with the upregulation of A1‐related gene expression (Figure 9D,E) and the pro‐inflammatory cytokines (Figure 9F,G). Moreover, the RPS3 knockdown in KCs and liver effectively abolished these TNF‐α KEVs and 1 dpi LEVs‐induced effects on the activation of astrocytes, resulting in a reduction in C3‐positive percentage (Figure 9A–C), A1‐related gene expression (Figure 9D,E), and the pro‐inflammatory cytokines (Figure 9F,G). Importantly, reversal of these effects by exogenous RPS3 supplementation confirms their specific dependence on RPS3. (Figure S6A–D). These data indicated that the LEVs promoted astrocyte polarization into A1 reactive astrocytes through RPSs.

Next, we injected PKH‐26‐labeled KEVs^GFP‐RPS3^ into SCI rats, and immunostaining with GFAP revealed that these labeled KEVs were taken up by GFAP‐positive astrocytes (Figure 9H). Subsequently, we knocked down RPS3 expression in the liver by injection of AAV8‐shRPS3, leading to a reduction of C3 expression in GFAP‐positive astrocytes at lesion sites (Figure 9I,J). This indicated that the liver‐derived RPS3 plays a key role in the activation of astrocytes.

Given the critical role of astrocyte polarization in regulating the spinal cord microenvironment after SCI and its impact on NSCs differentiation [12], we investigated whether LEVs‐stimulated astrocytes could influence NSC fate. We collected the conditioned medium (CM) from RPS3‐treated astrocytes (RPS3‐treated ACM) and added it to NSCs (Figure S7A). Immunostaining revealed that the addition of RPS3‐treated ACM markedly reduced the proportion of oligodendrocytes and neurons, compared to ACM‐treated NSCs (Figure S7B–D). Subsequently, we treated NSCs with the CM derived from KEVs‐treated astrocytes (KEVs‐treated ACM), TNF‐α KEVs‐treated astrocytes (TNF‐α KEVs‐treated ACM), and TNF‐α‐KEV^siRPS3^‐treated astrocytes (TNF‐α‐KEV^siRPS3^‐treated ACM) (Figure S7E). Compared to KEVs‐treated ACM, TNF‐α KEVs‐treated ACM strongly suppressed NSC differentiation into oligodendrocytes and neurons, whereas RPS3 depletion abolished this inhibitory effect (Figure S7F–H). Similarly, CM from LEVs‐treated astrocytes (LEVs‐treated ACM), 1 dpi LEVs ‐treated astrocytes (1 dpi LEVs‐treated ACM) and 1dpi LEVs^shRPS3^‐treated astrocytes (1dpi LEVs^shRPS3^‐treated ACM) were evaluated (Figure S7I). We found that 1 dpi LEVs‐treated ACM markedly decreased oligodendrocyte and neuronal differentiation, an effect that was reversed by knockdown of RPS3 in liver‐derived EVs (Figure S7J–L). These findings suggested that the RPS3 enriched LEVs or KEVs‐ promote an astrocytic phenotype that suppresses NSC differentiation into oligodendrocytes and neurons after SCI.

### 2.7 RPS3 Knockdown in the Liver Enhanced the Axonal Regeneration and Remyelination

**FIGURE 10:**
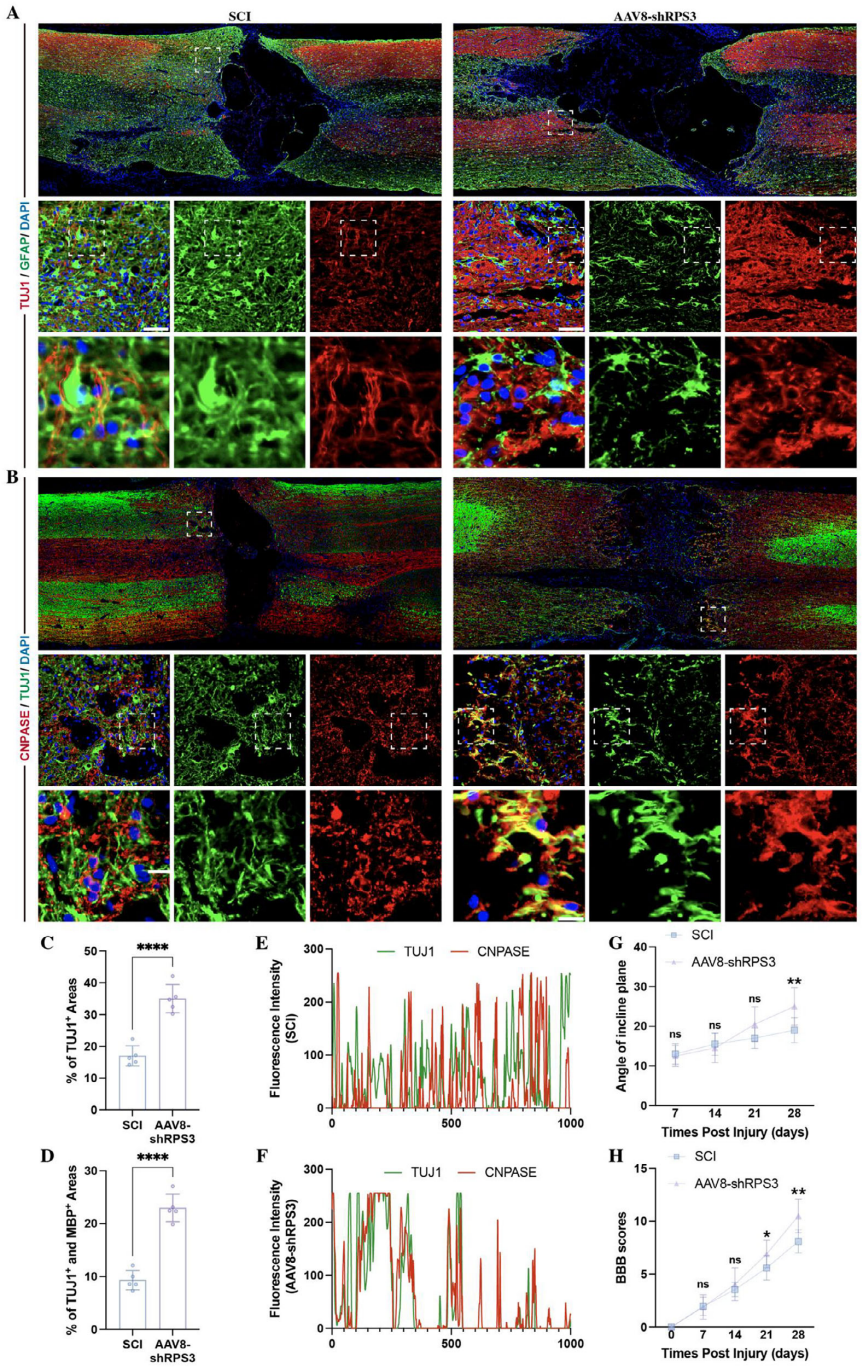
AAV8‐shRPS3 injection enhanced the axonal regeneration and remyelination. (A–D) Immunostaining detecting the expression of TUJ1‐positive neuronal neurite and TUJ1‐ and MBP‐double positive remyelinated axon in spinal cord from SCI rats and AAV8‐shRPS3 injected SCI rats (*n* = 5; scale bars, 100 µm). (E,F) Quantification of fluorescence intensity of TUJ1 and MBP at lesion sites. (G,H) Assessment of neurological function in SCI rats and AAV8‐shRPS3 injected SCI rats by BBB scores (G) and inclined plane test (H) (*n* = 10). All data are presented as the mean ± standard deviation (SD). ns, *p*>0.05, ^*^ *p* <0.05, ^**^ *p*<0.01, ^***^ *p*<0.001, ^****^ *p* <0.0001.

To investigate whether depletion of RPS3 in the liver could affect regeneration and remyelination of axons, immunostaining was performed at week 4 postinjury. Compared with SCI rats, the AAV8‐shRPS3‐injected rats significantly increased the TUJ1‐positive neuronal axon areas within the GFAP‐positive astrocytic scar boundary (Figure 10A,C). Moreover, the proportion of TUJ‐ and MBP‐positive remyelinated axons was increased in the injected rats (Figure 10B,D–F), indicating that the target depletion of RPS3 in the liver enhanced the axonal regeneration and remyelination. In line with the histological data, AAV8‐RPS3‐treated mice exhibited an improvement in the BBB scores (Figure 10G) and inclined‐plane tests (Figure 10H), indicating better neurological function recovery.

### 2.8 Elevated RPS3 in Plasma EVs Correlated With Severity and Poor Neurological Recovery Following SCI

**FIGURE 11:**
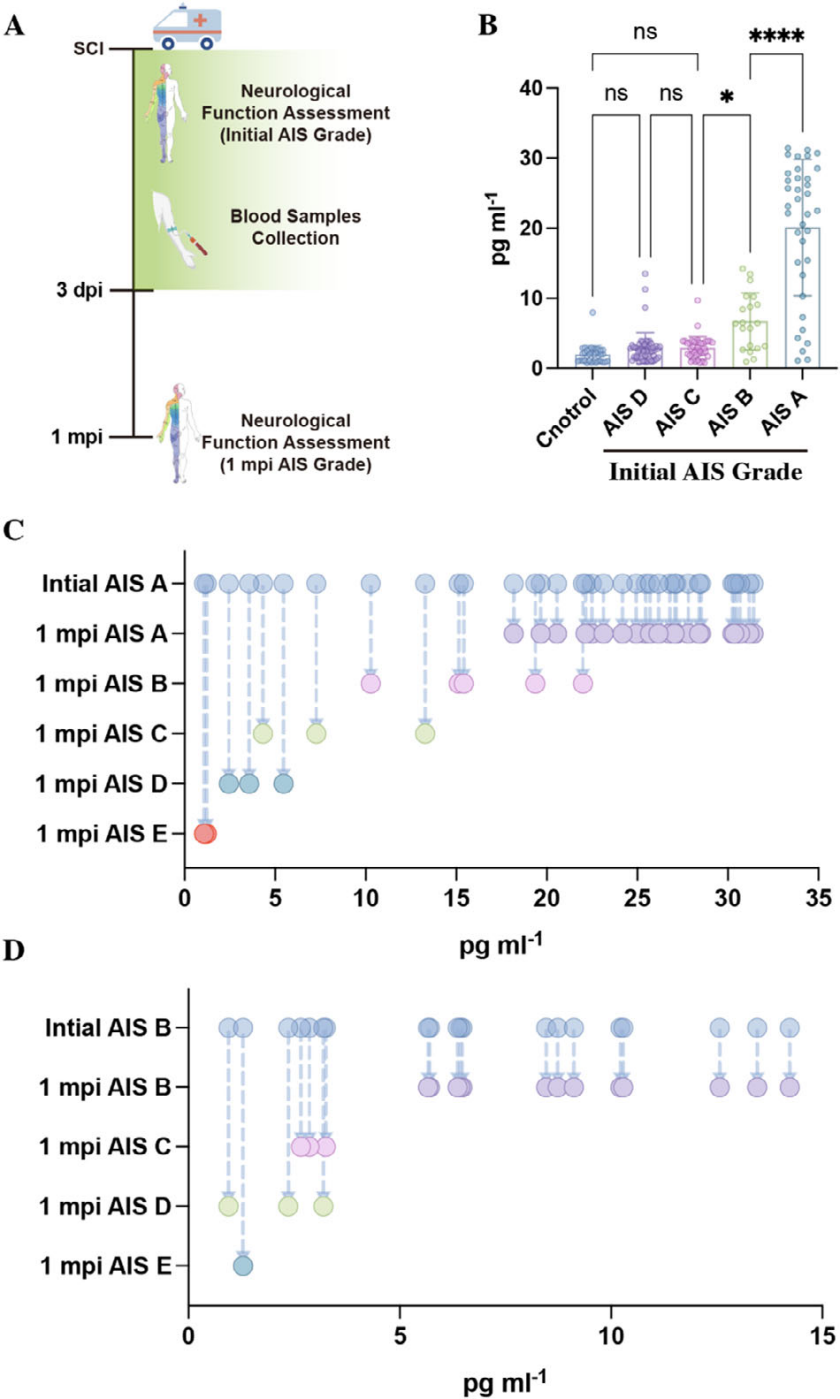
Elevated RPS3 in plasma EVs correlated with severity and poor neurological recovery following SCI. (A) Schematic of the time points of neurological assessment and blood sample collection. (B) Detection of RPS3 within PEVs from different initial AIS grade patients by ELISA. (C,D) Re‐assessing the AIS grade of initial AIS A (C) and AIS B (D) patients at 1 mpi. All data are presented as the mean ± standard deviation (SD). ns, *p*>0.05, ^*^ *p* <0.05, ^**^ *p*<0.01, ^***^ *p*<0.001, ^****^ *p* <0.0001.

To investigate whether RPS3 was associated with the degree of neurological impairment, we collected EVs from the plasma of SCI patients, and neurological function was evaluated on admission (within 3 days, initial AIS grade) (Figure 11A). SCI patients were divided into AIS grades A‐D. The clinical characteristics of SCI patients were illustrated in Figure S8 Measurement of RPS3 concentration in PEVs revealed significantly increased RPS3 expression in AIS A and AIS B patients compared to control groups and AIS C–D patients (Figure 11B), indicating an association between circulating RPS3 levels and neurological impairment severity. To further investigate whether the initial PRS3 levels in PEVs could predicted neurological recovery, we re‐assessed the neurological function at 1 month post‐SCI (1 mpi AIS grade) finding that, in initial AIS A (Figure 11C) or AIS B patients (Figure 11D), those exhibiting one or more AIS grade improvement had significantly lower RPS3 in PEVs compared to patients showing no functional recovery. This suggests that an early increase in RPS3 within PEVs predicts worse neurological outcome.

## 3 Discussion

In this study, we revealed a liver‐to‐spinal cord communication mediated by EVs whereby SCI activates hepatic KCs to release EVs loaded with RPS3. These LEVs are transported to the injury epicenter and mediate NSC differentiation and astrocyte polarization, thereby deteriorating the local regenerative microenvironment following SCI.

### 3.1 A Liver‐Spinal Cord Communication Pathway Influences Local Neural Recovery

Neurotrauma involves multicellular EV‐mediated crosstalk that plays roles in regulating the inflammatory process and nerve regeneration. Emerging evidence has found that beyond local communication, EVs carrying cell‐specific signaling cargoes play a key role in the inter‐organ communication network, inducing bidirectional blood‐borne EV crosstalk between the CNS and peripheral organs [10]. Following CNS injury, neuronal, microglial, and astrocytic markers were found to be expressed in plasma EVs, confirming that CNS‐shed EVs were leaking into circulation [32, 33, 34]. These EVs, which are transported into peripheral systems, induce dysfunction or failure in multiple organs. The liver plays a pivotal role in coordinating systemic organ functions and maintaining physiological homeostasis through bidirectional communication with other organs [35, 36]. It has been reported that CNS trauma triggers the liver to undergo a pro‐inflammatory transformation, known as the hepatic acute phase response [10].

In the present study, we identified a reverse liver‐to‐spinal cord communication pathway, with the liver as a critical remote organ dynamically influencing the CNS regenerative capacity after trauma. We found that LEVs were transported into lesion sites and directly taken up by NSCs and astrocytes. The bioeffects of LEVs were altered by SCI, showing a significant inhibition of NSC differentiation into neurons and oligodendrocytes, and a promotion of astrocyte polarization into neuroinflammatory reactive astrocytes. This indicated that the cargoes within LEVs were altered due to the hepatic acute phase response and led to detrimental bioeffects on local nerve regeneration.

### 3.2 The Role of KCs in the Liver‐Spinal Cord Inter‐Organ Communication Following SCI

The liver, functioning as an endocrine organ, secretes a variety of substances that influence the activities of other body organs [36, 37, 38, 39, 40]. KCs are local liver macrophages that play an important role in sustaining liver function. Due to their strategic location within liver sinusoids, KCs are rapidly activated in response to external stimuli and amplify systemic inflammatory responses by releasing pro‐inflammatory cytokines to adjacent cells and into the circulation [41]. We found that pre‐treatment with clodronate, which depleted KCs in the liver, effectively decreased the RPS3 release from the liver and led to a reduction of RPS3 expression at lesion sites, preventing the activation of NF‐κB signaling. It highlights the important role of KCs in the liver‐spinal cord interorgan communication. Furthermore, we found the pro‐inflammatory stimulation markedly promoted the enrichment of RPS3 within KEVs, enhancing the KEVs' effects on inhibiting NSC differentiation into neurons and oligodendrocytes and promoting astrocyte polarization into neuroinflammatory types. Moreover, the depletion of RPS3 in KCs markedly attenuated these KEVs‐related effects. These findings suggest that activated KCs are the primary cellular source of RPS3, highlighting the critical role of KCs in the liver‐spinal cord interorgan communication.

Our findings also uncover an intra‐hepatic cellular cascade that amplifies the liver's response to SCI. We observed a distinct temporal pattern of RPS3 induction: a rapid upregulation of RPS3 in CD68^+^ KCs occurring as early as 6 h post‐injury, followed by a delayed but significant increase in CD68^−^ cells predominantly albumin‐positive hepatocytes, which peaked at 1 and 3 dpi. Depletion of KCs via clodronate markedly abolished RPS3 upregulation in hepatocytes provides compelling evidence that activated hepatic macrophages are the initiators of this cascade. This was further corroborated by our in vitro experiments, wherein the conditioned medium from TNF‐α‐stimulated KCs was sufficient to induce RPS3 enrichment in hepatocyte‐derived EVs, which subsequently impaired NSC differentiation into oligodendrocytes and neurons.

A key question arising from our findings is how the initial signal originating from the SCI site that activates hepatic KCs. Although the present study did not directly identify the primary trigger, several lines of evidence point toward a plausible mechanism. It has been reported that CNS injury, including SCI, induces a rapid release of pro‐inflammatory cytokines (e.g., TNF‐α, IL‐1β, IL‐6) [10, 15, 27] and EVs into the circulation [32, 33, 34], which are prime candidates for inducing the activation of KCs in liver. This notion is strongly supported by our in vitro data showing that pretreatment of KCs with TNF‐α or IL‐6 markedly enhances RPS3 enrichment in KC‐derived EVs. Taken together, these data suggest a compelling feedback loop: SCI leads to the release of inflammatory mediators into the bloodstream, which activate KCs to promote a hepatic response, producing RPS3‐enriched EVs. These EVs, in turn, are transported back to the spinal cord lesion, exacerbating local neuroinflammation and inhibiting repair, thereby potentially sustaining or amplifying the initial systemic inflammatory signal.

### 3.3 RPS3 Functions as a Key Mediator in the Liver‐Spinal Cord Axis

Several lines of evidence support that RPS3 plays a key role in this inter‐organ communication. First, MS‐based proteomics revealed a significant enrichment of RPS3 within plasma‐EVs from SCI patients. In line with these findings, RNA‐sequence analysis on post‐SCI liver also found the upregulation of PRS3 gene expression. Subsequent experiments confirmed a marked upregulation of RPS3 within EVs derived from the liver at 1 and 3 dpi; Second, the treatment of postinjury LEVs promoted P‐P65 expression and its translocation into the nucleus, accompanied by the upregulation of its downstream gene, proving NF‐κB activation. Furthermore, these LEVs‐related bioactivates were effectively attenuated by the addition of an NF‐κB inhibitor. Third, targeted depletion of RPS3 in the liver decreased the RPS3 expression and inhibited the NF‐κB activation at lesion sites. In vitro experiments revealed that RPS3 depletion in KCs attenuated their EVs‐related effects on regulating NSC differentiation and astrocyte polarization. Fourth, liver‐specific FLAG‐tagged RPS3 overexpression revealed that the presence of FLAG^+^ liver‐derived RPS3 in Nestin^+^ NSCs and GFAP^+^ astrocytes, confirming direct transfer of RPS3 from the liver to the spinal cord lesion following SCI.

Notably, these data could not conclude that the alterations of RPS3 at lesion sites are exclusively due to hepatic RPS3. Following SCI, the activated liver not only generates pro‐inflammatory cytokines and chemokines but may also amplify inflammatory signaling systemically by engaging systemic immune system [10, 15]. Thus, we fully acknowledge that the liver may not be the sole source of RPS3 upregulation at the lesion site. It is plausible that SCI, or the liver in response to SCI, activates macrophages in other extraspinal organs or the peripheral circulation, which in turn contribute to the elevated RPS3 levels at lesion sites. Nevertheless, the marked reduction in RPS3 at the lesion site following liver‐specific RPS3 knockdown underscores the central role of the liver in the systemic RPS3 response after SCI, even if other organs or cell types participate in this process.

### 3.4 RPS3‐Enriched EVs Have Dual Significance as a Clinical Biomarker and a Stable Delivery Vehicle

Proteins and molecular material can be packed into EVs and transported to the peripheral circulation at concentrations of > 10^9^ vesicles/mL [42], providing ample biological specimens for diagnostic and prognostic biomarkers [42, 43, 44]. In clinical samples of SCI patients, we found the early enrichment of RPS3 in PEVs strongly indicated severe SCI and worse neurological recovery. Besides the alteration of cargoes within EVs, the lipid bilayer membrane of EVs protects cargoes from degradation, offering enhanced stability compared to free circulating molecules [10, 42]. Thus, EVs are designed as natural nanoscale transport vesicles for the effective delivery of proteins, microRNAs, siRNAs, etc [45, 46, 47, 48]. In the present study, we found that compared with the injection of KEVs, the injection of non‐EVs KCM failed to transport exogenous GFP‐RPS3 to lesion sites, highlighting that the lipid bilayer membrane of EVs plays a critical role in the long‐distance delivery of RPS3.

Taken together, we identified a previously unrecognized liver‐to‐spinal cord communication pathway that actively impedes recovery after SCI. We demonstrated that SCI activates hepatic KCs to release EVs enriched with RPS3, which are transported to lesion sites and preferentially taken up by NSCs and astrocytes. These RPS3‐enriched LEVs promote the activation of NF‐κB signaling, leading to a cascade that suppresses NSC differentiation into neurons and oligodendrocytes while promoting polarization of astrocytes into neuroinflammatory phenotypes, thus creating an inhibitory microenvironment for regeneration.

## 4 Experimental Section

### 4.1 NSC Culture and Differentiation

NSCs were isolated according to our previous protocols [12, 49]. Briefly, cells dissected from the spinal cord were expanded as suspended neurospheres for 7 days in serum‐free Dulbecco's Modified Eagle Medium/Nutrient Mixture F‐12 (DMEM/F12) (Gibco, MA, USA) supplemented with 2% B27 supplement (Gibco, MA, USA), 20 ng mL^−^^1^ epidermal growth factor (Gibco, MA, USA), and 10 ng mL^−^^1^ basic fibroblast growth factor (Gibco, MA, USA). Half the volume of the medium was replenished with fresh NSC medium every 3 days. Following the 7‐day suspension culture, neurospheres were enzymatically dissociated into single cells, which were then plated on poly‐D‐lysine‐coated coverslips at a density of 1 × 10^5^ cells per well. Cells were maintained adherently in NSC differentiation medium containing DMEM/F12 and 2% B27 supplement, with the medium replaced every 3 days.

### 4.2 Primary Astrocyte Culture

Primary astrocytes were isolated from spinal cords of 3‐day‐old rats using established protocols [50]. Cells were seeded in Minimum Essential Medium (MEM; Thermo Fisher Scientific, USA) containing 10% fetal bovine serum (FBS) (Gibco, USA) and 1% antibiotics, then cultured for 7 days with medium changes every three days. After reaching confluence, the cells were shaken overnight at 280 rpm at 37°C to remove microglia and oligodendrocytes, the adhering cells were collected as astrocytes. Three passaged astrocytes were used for further experiments.

### 4.3 Primary KC Culture and Transfection

KCs were isolated by collagenase digestion and differential centrifugation according to a previous study [24]. Briefly, the liver was perfused via the portal vein at 37°C for 10 min using calcium‐ and magnesium‐free Hank's Balanced Salt Solution, followed by an additional 10‐min perfusion with Hank's Balanced Salt Solution containing 0.02% collagenase IV (Sigma). Subsequently, the liver was digested and cut into fragments within the collagenase solution. To remove parenchymal cells, the cell suspension was filtered through a sterile 100 µm nylon cell strainer. The non‐parenchymal cells‐enriched supernatant was collected, washed with buffer, and centrifuged at 500 ×g for 5 min. The cell pellets were resuspended in buffer and subjected to gradient centrifugation on a discontinuous Percoll gradient (25%/50%) at 1800 × g for 15 minutes at 4°C. KCs were collected by centrifugation at 650 g for 7 min and resuspended in buffer. The isolated KCs were cultured in Roswell Park Memorial Institute 1640 medium (Sigma) containing 10% FBS (Gibco, USA) and 1% antibiotics. Following a 30‐min culture, nonadherent cells were removed.

To achieve the knockdown of RPS3, small‐interfering RNAs (siRNAs) targeting RPS3 were purchased from Invitrogen. The green fluorescence protein (GFP)‐RPS3 overexpression plasmid was synthesized by Zebrafish Biotech. Lipofectamine 2000 reagent (Invitrogen, USA) was used for cell transfection. The transfection process was carried out in serum‐free medium with the addition of siRNAs according to the manufacturer's instructions. Following transfection, cells were cultured for 24 h, followed by polymerase chain reaction (PCR) detection to evaluate the expression levels of the target genes.

### 4.4 KC‐Derived EVs Collection

The procedure for KC‐derived EVs collection was described in our previous study [12]. Briefly, before collecting EVs, the KCs' culture medium was switched to serum‐free medium. Following 24 h culture, the supernatant was collected for ultracentrifugation to obtain EVs. The collected EVs were resuspended in phosphate‐buffered saline (PBS) (Gibco, USA) and stored at −80°C.

### 4.5 LEVs Collection

The procedure for LEVs collection was performed according to previous protocols [35]. Briefly, isolated liver lobes from SCI rats were immediately transferred to oxygenated M199 medium containing 10% FBS and 1% antibiotics. These collected liver lobes were cut into 300‐µm slices, washed in PBS, and subsequently subjected to a 1‐h recovery incubation at 37°C in oxygenated M199 containing 1% antibiotics. Following an additional PBS wash, slices were incubated with oxygenated EX‐CELL protein‐free culture medium (Sigma, USA) for 16 h. The conditioned medium was collected and subjected to ultracentrifugation to obtain LEVs. Transmission electron microscopy, dynamic light scattering, and western blotting for TSG101, CD9, and CD63 were performed to identify the EVs.

### 4.6 EVs Tracking

The collected EVs were fluorescently labeled using PKH26 (Sigma, USA) following the manufacturer's protocol. Briefly, EVs were incubated with 4 µL of PKH26 dye diluted in 1 mL of Diluent C for 4 min at room temperature. These PKH26‐labeled EVs were then recollected by ultracentrifugation at 100 000 × g for 70 min.

### 4.7 MS for Proteomics

Blood samples were obtained from three association impairment scale (AIS) A patients within 3 days of cervical SCI. The exclusion criteria for SCI patients included: presence of other polytrauma; evidence or history of neurological diseases (e.g., Parkinson's disease, Alzheimer's disease, amyotrophic lateral sclerosis, etc.) that might affect the evaluation of neurological function; pre‐existing liver diseases; failure to obtain a blood sample within 3 days postinjury; and long‐term corticosteroid use (over 30 days). Three healthy controls, matched for age and gender to the SCI patients, were enrolled. Healthy controls were subject to similar exclusion criteria, including any history of neurological or liver diseases, as well as prolonged corticosteroid use. Ultracentrifugation was performed to collect the plasma‐derived EVs. Following ultra‐trace samples preparation and peptide‐level quantification, each sample was mixed with an iRT standard (Thermo Fisher Scientific, USA) at a volume ratio of 1:20 (iRT: test sample). A timsTOF MS platform (Bruker, Germany) operating in data‐independent acquisition mode was used to obtain raw data at the Metabolomics Laboratory of OE biotech (Shanghai, China). The raw data were then subjected to direct data‐independent acquisition spectral library matching, extraction of quantitative information, and subsequent statistical analyses.

### 4.8 Blood Sample Collection and Neurological Examination of SCI Patients

Blood samples were obtained from patients suffering from cervical SCI within 3 days and undergoing medical treatment in the Department of Spinal Surgery of The First Affiliated Hospital of Anhui Medical University. Written informed consent was provided by each participant before enrollment in this study. By ultracentrifugation, the plasma EVs were collected for RPS3 measurement using the RPS3 ELISA kit according to the manufacturer's instructions. The American Spinal Injury Association impairment scale (AIS) [51] was used to evaluate neurological function at the time of admission (within 3 days) and on day 7 post‐SCI by two neurosurgeons. This study was approved by the Ethics Committee of The First Affiliated Hospital of Anhui Medical University (NO.5101460; NO. 2024572).

### 4.9 An Animal SCI Model and Administration

A severe crush SCI model was performed on female SD rats aged 6–8 weeks from the Animal Facility of Anhui Medical University according to our previously established protocols [14]. SD rats were maintained in clean and warm cages under controlled conditions (22°C–25°C, 12 h light/dark cycle) with an adequate supply of water and food. All procedures adhered to 3R principles and were approved by the Institutional Animal Ethics Committee (LLSC20241680). After randomization and anesthesia, a T10 laminectomy was performed to expose the spinal cord, followed by a bilateral compression for 5 s with a 0.5 mm tip‐ Dumont forceps to induce a severe crush SCI model. For tissue preparation, rats were fixed via transcardiac perfusion with ice‐cold PBS, followed by 4% paraformaldehyde. The rats, which were divided into clodronate groups, received continuous intravenous injection of clodronate (5 mg of clodronate per 100 g of rat weight) from day 3 before SCI to day 3 post‐injury. For hepatocyte‐specific RPS3 knockdown, adeno‐associated virus 8 (AAV 8) carrying short hairpin RNA was designed by Zebrafish Biotech. The rats were administered 2 × 10^11^ vg/mL of AAV8‐shRPS3 by tail vein injection at day 7 before SCI. The locomotor functional deficits were measured blindly using the Basso–Beattie–Bresnahan (BBB) scores and the inclined plane test at different time points by two independent individuals.

### 4.10 Tissue Preparation

For immunostaining, spinal cord tissues were fixed in 4% paraformaldehyde (4°C, 24 h) and longitudinally sectioned (4 µm) through the lesion epicenter using a Leica RM2135 electric slicer (Leica, Germany). For observation of PKH26‐labeled EVs, the SCI rats were administered 25 µL of PKH‐26‐labeled EVs through tail vein injection. Then, the spinal cords were collected, fixed with 4% paraformaldehyde for 24 h, followed by incubation with 10%, 20%, or 30% sucrose solution at 4°C. A Leica CM3050 electric slicer was used to section the spinal cord into 4 µm longitudinal slices.

### 4.11 Immunofluorescence Staining

Neurospheres of NSCs were enzymatically dissociated into single cells and seeded on the glass‐bottom dishes for 7 days of culture. The prepared cells or tissues were incubated overnight at 4°C with primary antibodies: mouse anti‐CNPase for oligodendrocytes (1:200; Abcam, United Kingdom), rabbit anti‐glial fibrillary acidic protein (GFAP) for astroglia (1:1000; Abcam, United Kingdom), rabbit anti‐myelin basic protein (MBP) for oligodendrocytes (1:1000; Abcam, United Kingdom), rabbit anti‐neuron‐specific class III beta‐tubulin (TUJ1) for neurons (1:1000; Abcam, United Kingdom), mouse anti‐phosphorylated P65 (P‐P65) (1:1000; Abcam, United Kingdom), and RPS3(1:200; Proteintech, USA). The secondary antibodies used were Cy3 (1:50; Elabscience, China) and Alexa Fluor 488 (1:50; Elabscience, China), and DAPI was used to counterstain nuclei. Images were acquired using a Leica DM‐6B fluorescence microscope (Leica, Germany). The positive percentage of areas and colocalization was analyzed by ImageJ. To count the positive cells, 10–15 fields with a total of 500–1000 cells from 5 individuals were selected to quantify the percentage of positive cells.

### 4.12 Immunoprecipitation

NSCs were washed with PBS and lysed on ice using immunoprecipitation lysis buffer supplementary with 2 nm phenylmethylsulfonyl fluoride (PMSF). The cell lysates were then centrifuged at 12 000 × g for 15 min at 4°C, and the supernatant was collected. The immunoprecipitates were harvested using Protein A/G Agarose beads. The beads were then collected by centrifugation and washed five times. The samples were finally separated by SDS‐PAGE and analyzed by western blotting.

### 4.13 Western Blot Assay

EVs were lysed in lysis buffer to extract proteins. A bicinchoninic acid assay kit (Thermo Fisher Scientific, USA) was used to quantify protein concentrations according to the manufacturer's protocol. Equal protein quantities were loaded and separated by sodium dodecyl sulfate‐polyacrylamide gel electrophoresis, followed by transfer onto polyvinylidene difluoride membranes. Membranes were blocked with 5% nonfat milk in Tris‐buffered saline containing 0.1% Tween‐20 for 1 h at room temperature. Primary antibody incubation was performed overnight at 4°C using anti‐CD 63(1:1000; Immunoway, USA), anti‐CD 9 (1:1000; Immunoway, USA), and anti‐TSG 101(1:1000; Immunoway, USA). After three 10‐min Tris‐buffered saline containing 0.1% Tween‐20 washes, membranes were incubated with secondary antibodies (1:5000 dilution, Elabscience, China) for 1 h at room temperature. Protein bands were detected using the Super‐Signal West Pico enhanced chemiluminescence reagent (Advansta, USA). Quantitative analysis of band intensities was performed using ImageJ.

### 4.14 RNA Isolation and Quantification

Total RNA was isolated from cultured cells using TRIzol reagent (Thermo Fisher Scientific, USA) according to the manufacturer's protocol. SuperScript III Reverse Transcriptase (Invitrogen, USA) was used to synthesize complementary DNA. Quantitative PCR (qPCR) was performed on a RealPlex2 thermal cycler (Eppendorf, Germany) with SYBR Green PCR Master Mix (Applied Biosystems, USA) and specific gene primers. Glyceraldehyde 3‐phosphate dehydrogenase was used as the reference gene for normalizing messenger RNA expression.

For RNA‐sequencing analysis, RNA was extracted from the liver at 1 and 3 days post‐injury (dpi). RNA sequencing and data analysis were performed by OE biotech (Shanghai, China). The procedure of RNA sequencing included sample quality control, library construction, and sequencing, and bioinformatic analysis. A qualified complementary DNA library was sequenced on an Illumina sequencing platform.

### 4.15 Statistical Analysis

All data are presented as the mean ± standard deviation (SD). Statistical analysis was evaluated by Student's *t*‐test, one‐way analysis of variance followed by Tukey's post hoc test for multiple comparisons, and two‐way analysis of variance followed by Bonferroni's test for multiple comparisons by using Prism 8.0. Mann–Whitney test and Kruskal–Wallis test were used for non‐parametric data. The *p*‐value <0.05 was regarded as a significant difference (ns *p*>0.05, ^*^ *p* <0.05, ^**^ *p*<0.01, ^***^ *p*<0.001, ^****^ *p* <0.0001). ImageJ (version 1.53a) was used for image analysis.

## Author Contributions

C.S., T.H., and J.Q contributed to the conception and design of the work; P.S. contributed to the research design, acquisition, and analysis of the data, and manuscript writing; T.H. contributed to the manuscript writing, analysis, and interpretation of the data; P.S., Z.W., Y.D., Y.F., S.B., D.W., F.H., and Z.W. contributed to the acquisition of the data and software used in the work; and T.H., Z.W., Y.L., and Y.W. contributed to the acquisition and analysis of the data. All the authors have read and approved the final manuscript.

## Conflicts of Interest

The authors declare no conflicts of interest.

## Supporting information




**Supporting File**: advs73718‐sup‐0001‐SuppMat.docx.

## Data Availability

All the data needed to evaluate the conclusions in the paper are presented in the paper and/or the Supplementary Materials. The authors declare that all supporting data and materials presented within this article and in the Data Supplement are available from the corresponding author by reasonable request.
