# Supplementary material for: RPS3‐Enriched Extracellular Vesicles Mediate Liver‐Spinal Cord Inter‐Organ Communication
Source: Adv Sci (Weinh). 2026 Jan 9;13(16):e17019. doi: 10.1002/advs.202517019 (PMC13042475; doi:10.1002/advs.202517019)
Supplement: Supplementary file 1 — Supporting File: advs73718‐sup‐0001‐SuppMat.docx. [file ADVS-13-e17019-s001.docx]

**
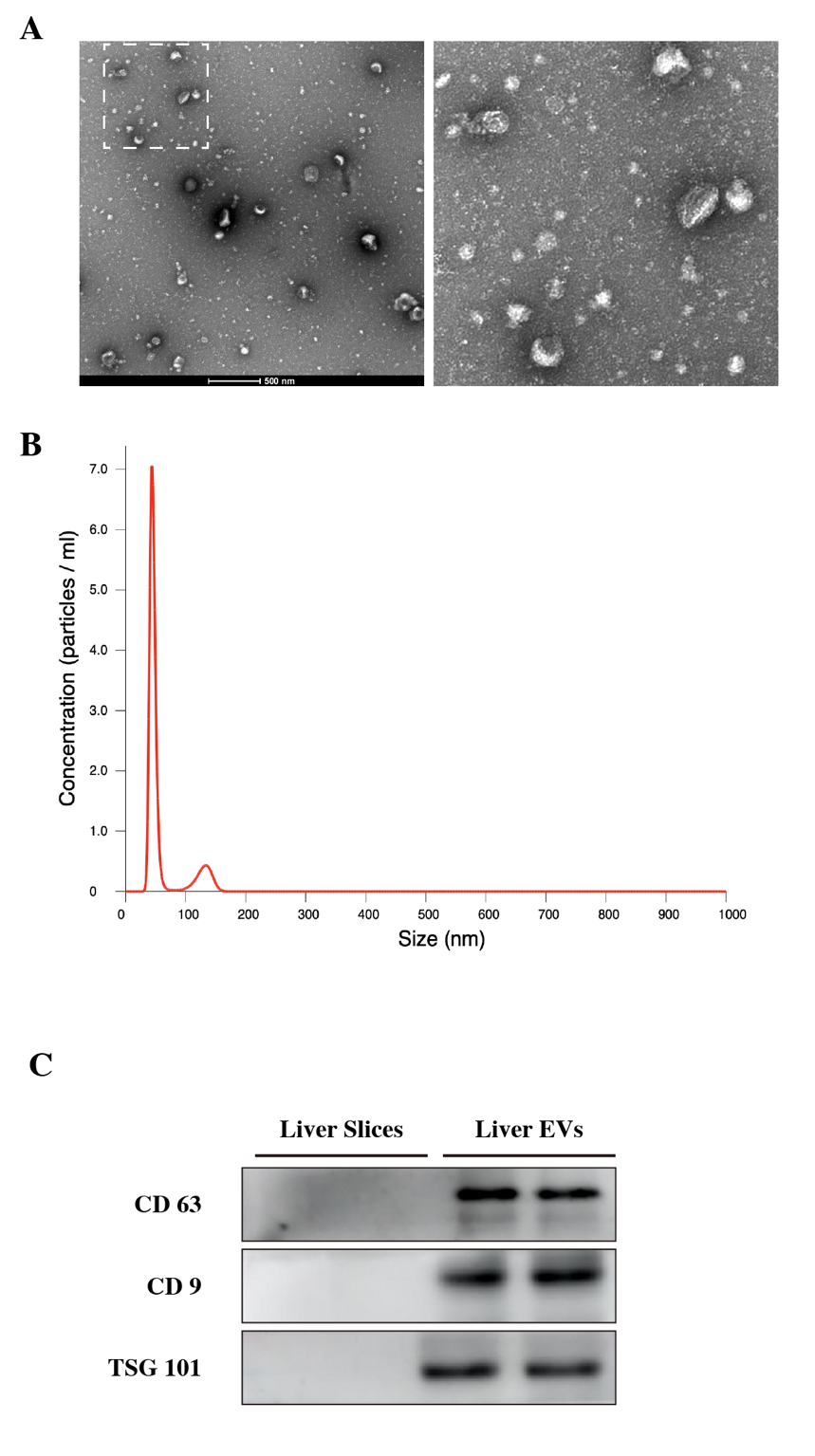
**

**Supplementary Figure.1 Identification of LEVs**

1. Transmission electron microscopy images of LEVs. B) Dynamic light scattering analysis of LEVs. C) Western blotting for CD 63, CD9 and TSG.


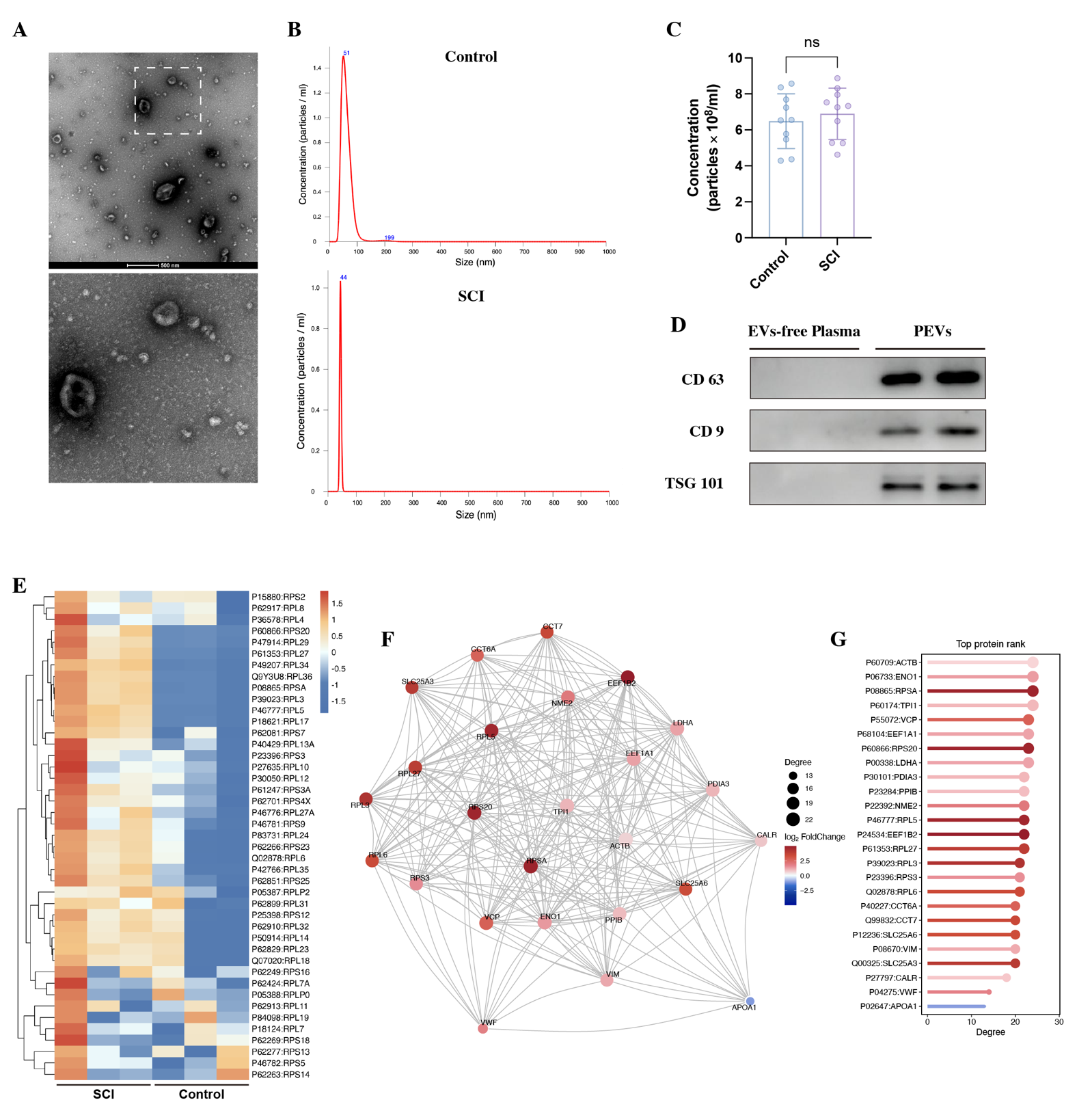


**Supplementary Figure.2 Identification and Bioinformatics Analysis of plasma EVs from SCI patients.**

A) Transmission electron microscopy images of PEVs. B, C) Dynamic light scattering analysis of PEVs. D) Western blotting for CD 63, CD9 and TSG. E) GSEA of gene expression. F, G) Network analysis of protein-protein interactions


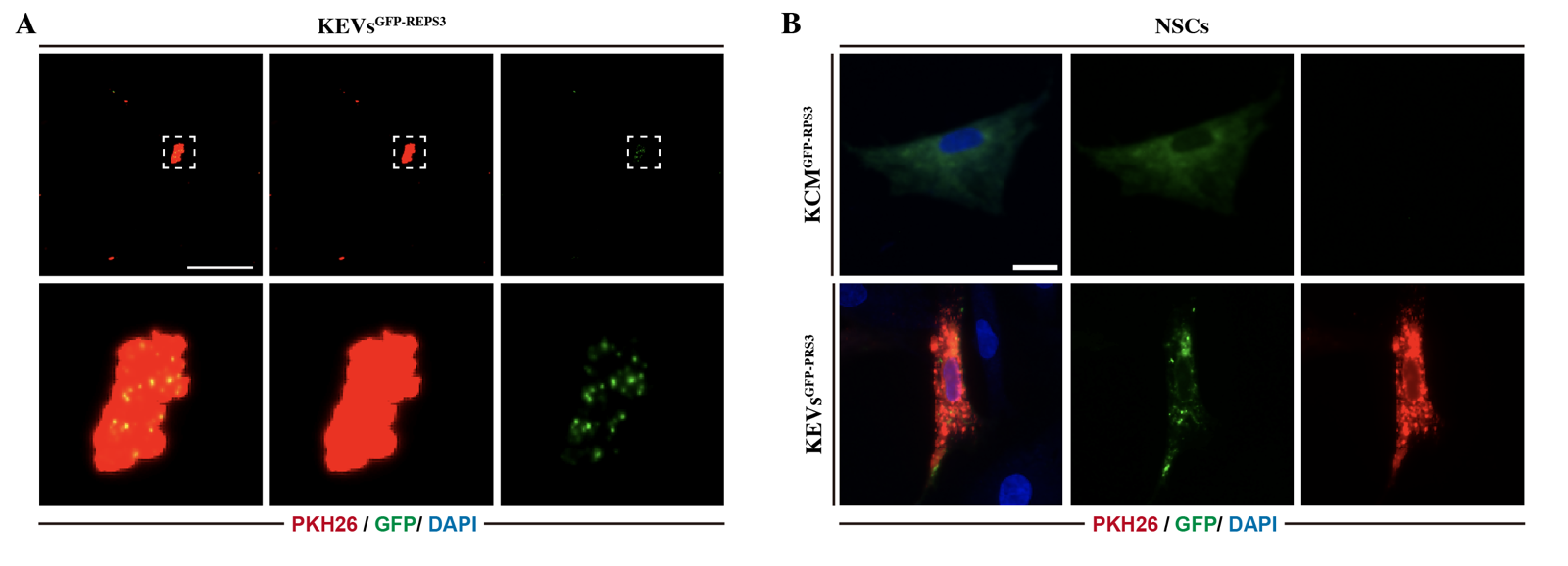


**Supplementary Figure.3 Tracing of KEVs^GFP-RPS3^ and KCMs^GFP-RPS3^**

A) Fluorescence microscopy showing the GFP-RPS3-transfected and PKH-26 labeled KEVs (scale bars, 100 μm). B) Fluorescence microscopy showing the expression of GFP-PRS3 (green) and PKH-26 (red) within NSCs following the addition of KCM^GFP-RPS3^ and PKH-26-labeled KEVs^GFP-RPS3^ (scale bars, 25 μm).


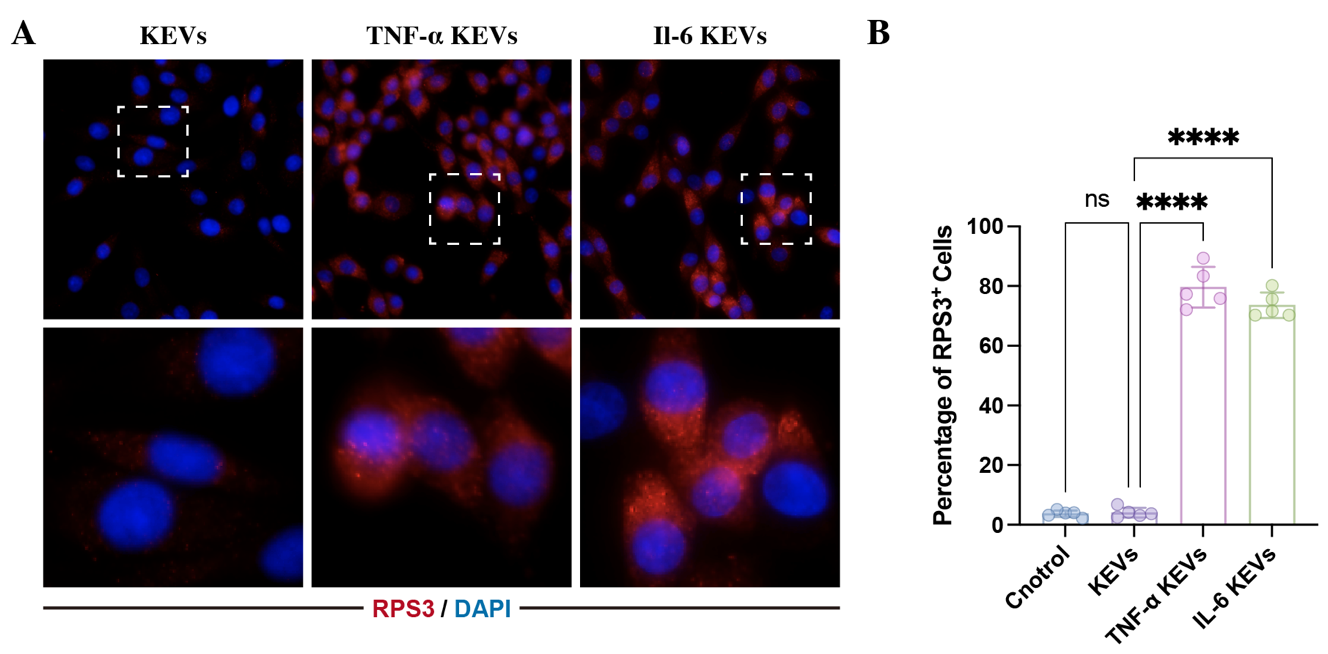


**Supplementary Figure.4 Pro-inflammatory cytokines promote RPS3 expression in KCs**

A) Immunostaining of RPS3 in TNF-α KEVs- or IL-6-KEVs-treated NSCs (n=5; scale bars, 50 μm). B) Quantification of the percentage of RP3S-positive cells. All data are presented as the mean ± standard deviation (SD). ns, P＞0.05; *, P < 0.05; **, P<0.01; ***, P<0.001; ****, P < 0.0001


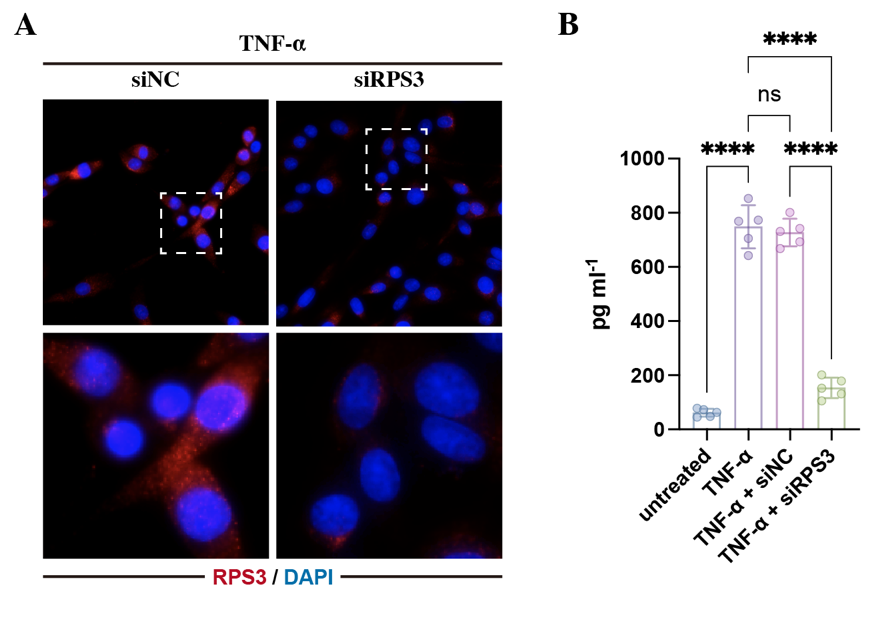


**Supplementary Figure.5 Validation of RPS3 knockdown in KCs**

A) Immunostaining of RPS3 confirming RPS3 knockdown in KCs. B) ELISA detecting RPS3 expression in TNF-α KEVs^siRPS3^(n=5). All data are presented as the mean ± standard deviation (SD). ns, P＞0.05; *, P < 0.05; **, P<0.01; ***, P<0.001; ****, P < 0.0001


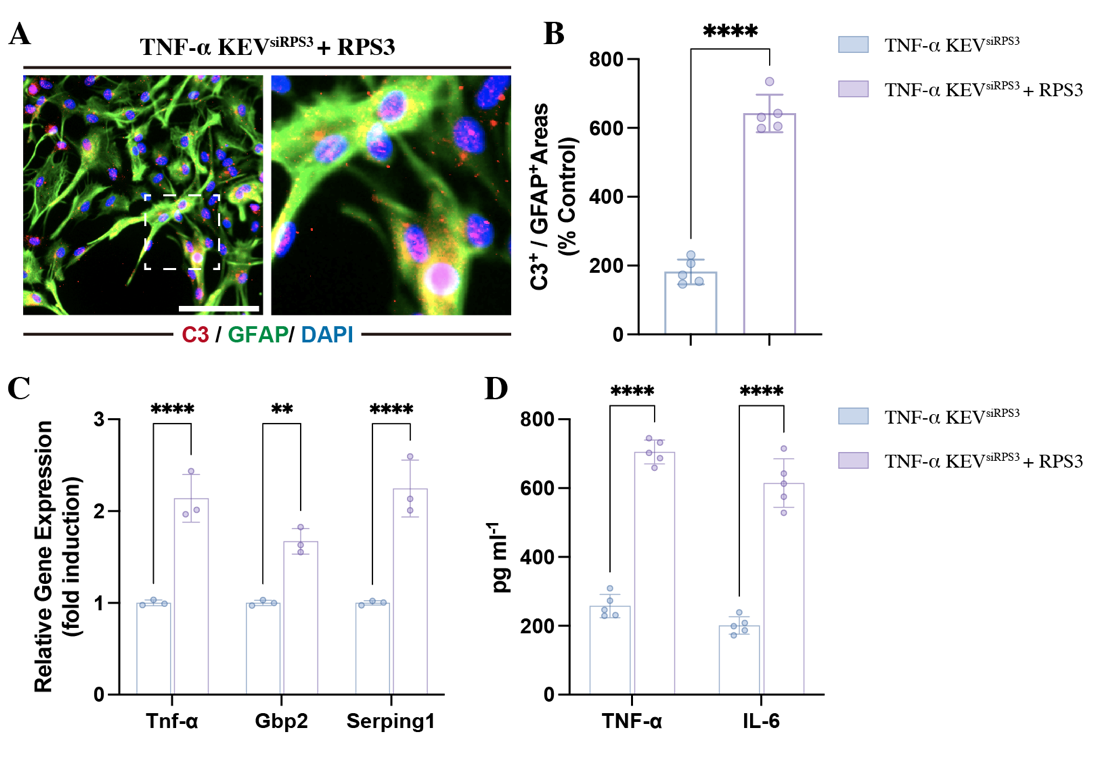


**Supplementary Figure.6 Exogenous RPS3 supplementation rescued the bioeffects of TNF-α KEVs^siRPS3^ on regulating astrocyte polarization.**

A-B) Immunostaining of C3 in GFAP-positive astrocytes treated with TNF-α KEVs^siRPS3^, with or without exogenous RPS3 supplementation (n=5; scale bars, 100 μm). C) RT-qPCR analysis of A1-related gene expression in astrocytes treated with TNF-α KEVs^siRPS3^, with or without exogenous RPS3 supplementation (n=3). D) ELISA detecting the expression of astrocyte-released pro-inflammatory cytokines following the treatment of TNF-α KEVs^siRPS3^, with or without exogenous RPS3 supplementation (n=5). All data are presented as the mean ± standard deviation (SD). ns, P＞0.05; *, P < 0.05; **, P<0.01; ***, P<0.001; ****, P < 0.0001


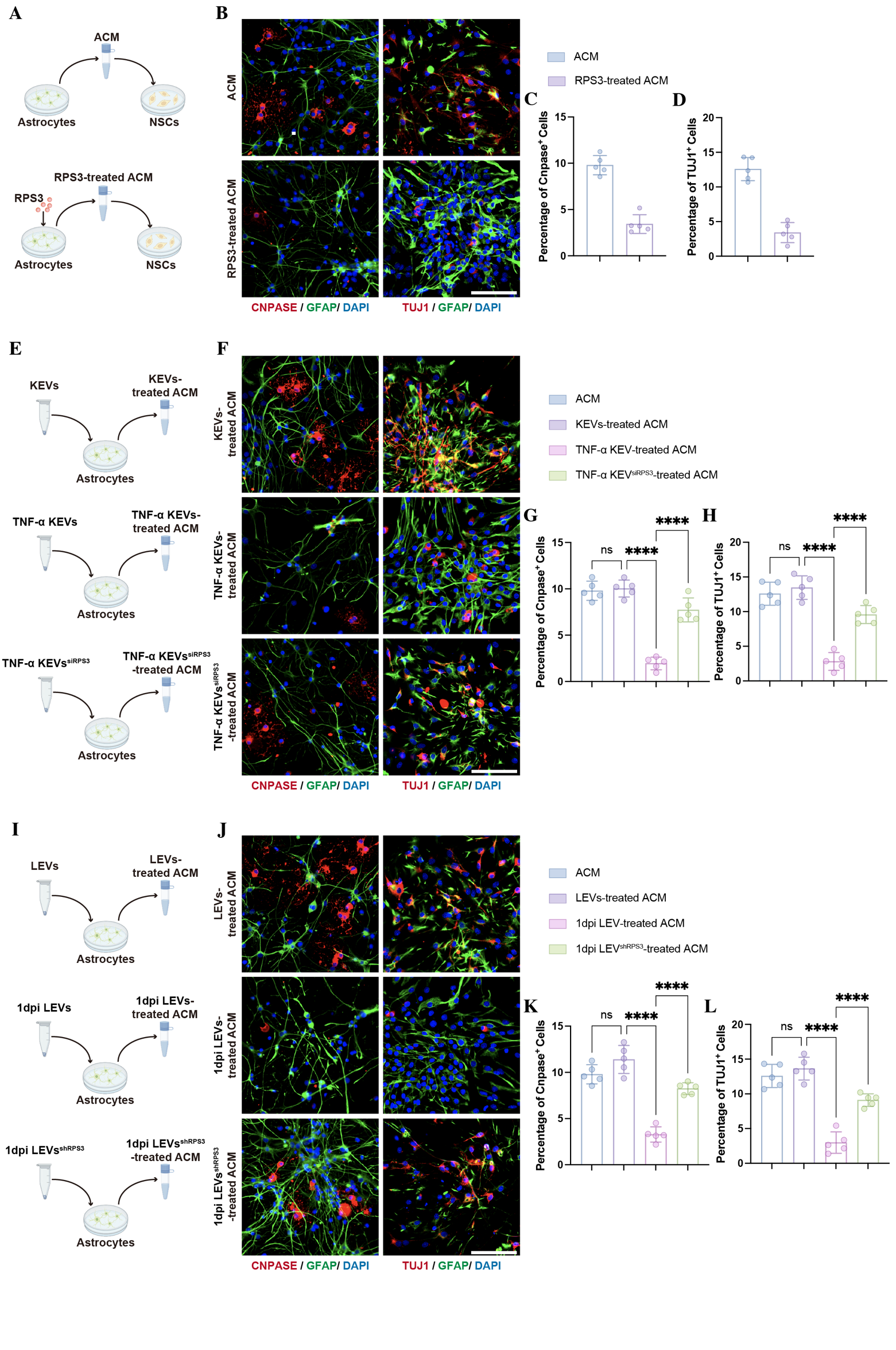


**Supplementary Figure.7 Livers/KC3-induced polarized astrocytes inhibit neuronal and oligodendrocytic differentiation of NSCs**

A) Schematic of RPS3-treated ACM collection for subsequent treatment of NSCs. B-D) Representative images and related quantification of the percentage of CNPase-positive and TUJ1-positive cells in NSCs that were treated with ACM or RPS-treated ACM for 5 days (n=5, scale bars, 100 μm). E) Schematic of KEVs-treated ACM collection for subsequent treatment of NSCs. F-H) Representative images and related quantification of the percentage of CNPase-positive and TUJ1-positive cells in NSCs that were treated with KEVs-, TNF-α KEVs-, or TNF-α KEVs^siRPS3^- treated ACM for 5 days (n=5, scale bars, 100 μm). I) Schematic of LEVs-treated ACM collection for subsequent treatment of NSCs. J-L) Representative images and related quantification of the percentage of CNPase-positive and TUJ1-positive cells in NSCs that were treated with LEVs-, 1 dpi LEVs-, or 1dpi LEVs^siRPS3^- treated ACM for 5 days (n=5, scale bars, 100 μm). All data are presented as the mean ± standard deviation (SD). ns, P＞0.05; *, P < 0.05; **, P<0.01; ***, P<0.001; ****, P < 0.0001


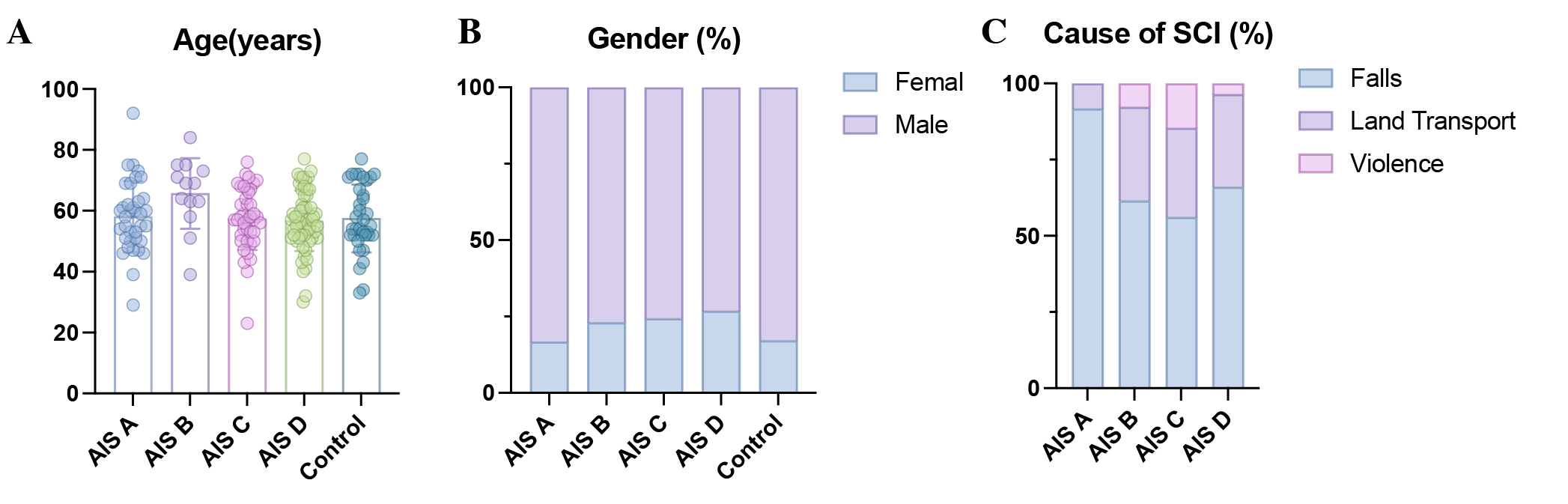


**Supplementary Figure.8 The clinical characteristics of SCI patients**
